# Association between maternal employment and the child´s mental health: a systematic review with meta-analysis

**DOI:** 10.1007/s00787-023-02164-1

**Published:** 2023-02-13

**Authors:** Marie Kopp, Marina Lindauer, Susan Garthus-Niegel

**Affiliations:** 1 Faculty of Medicine Carl Gustav Carus, Institute and Policlinic of Occupational and Social Medicine, Technische Universität Dresden, Dresden, Germany; 2 Institute for Systems Medicine (ISM) and Faculty of Medicine, Medical School Hamburg, Hamburg, Germany; 3 Department of Childhood and Families, Norwegian Institute of Public Health, Oslo, Norway

**Keywords:** Maternal employment, Child mental health, Systematic review, Meta-analysis

## Abstract

The recent rise in maternal workforce participation has led to more research regarding the role of maternal employment for (early) childhood mental health. This systematic review with meta-analysis covers new evidence on the association of both variables. A systematic literature search was conducted. Studies had to compare children 0–7 years of age on the basis of their mothers’ employment status, working amount, employment duration, i.e., how long the mother had been back at work after birth, or timing of return to work. Child mental health was operationalized as behavior problems and prosocial behavior. Narrative and meta-analytic syntheses of evidence were conducted. Maternal employment was associated with more conduct problems but less internalizing behavior problems and anxious/depressed behavior in children; full-time employment was linked to more externalizing behavior problems and more hyperactivity/inattention. Longer employment duration was related to less (internalizing) behavior problems and more prosocial behavior but also more externalizing behavior problems. Narrative syntheses indicated early maternal return to work to be associated with more child externalizing behavior problems and less prosocial behavior. Whether maternal employment is associated with child mental health strongly depends on both variables’ operationalization. Especially part-time employment, longer employment duration, and return to work only after the first year postpartum may be beneficial for child mental health. Practical implications pertain to an expanded offer of family leave and the endorsement of maternal employment after the first year postpartum. Here, factors that may buffer the negative associations with full-time employment warrant consideration.

**Supplementary Information:**

The online version contains supplementary material available at 10.1007/s00787-023-02164-1.

## Introduction

Mental health disorders often already appear during childhood and adolescence. Meta-analytic data provide a global prevalence rate of 13.4% in children up to age 18 years [1]. Inclusion of subclinical problems leads to even higher rates [2]. Mental health problems can already be observed in early childhood [3–5], where they are often operationalized as behavior problems (BP). Once developed, mental health problems are highly stable and often persist into adulthood [6], thereby being associated with adverse life outcomes, e.g., reduced long-term (mental) health [7, 8], suicidality [8], and delinquency [9]. Consequently, effective prevention and intervention strategies are highly warranted. Thus, research has aimed to identify variables associated with early mental health problems. Such factors are located in the child itself, e.g., its genetics [10, 11], sex [12, 13], and temperament [14], as well as in the child’s environment. Examples include family structure [15], parental mental health [12, 13, 15], and socio-economic status [13, 16]. Due to a substantial rise in maternal workforce participation in recent decades [17], research also focused on the role maternal employment plays in children’s early mental health.

### Linking maternal employment and child mental health theoretically

Several disciplines address a potential association between maternal employment and child mental health, i.e., developmental psychology, sociology, and economics [18, 19]. The household economics framework for instance postulates that families tend to maximize their well-being based on two resources, i.e., time and money. Parents face the decision whether well-being is maximized by mothers working or staying at home. While maternal employment leads to increased financial resources that can be used to purchase goods and services, e.g., child care, stay-at-home mothers may spend more time with their children, providing attention and emotional support. Maternal employment could lead to poorer child mental health in case goods and services purchased do not sufficiently compensate for lost parental care [see 20].

In light of sociological theories, the mediating role of maternal health seems of importance. The scarcity hypothesis for instance assumes that as time and energy are limited, one could not have enough energy to fulfil several roles equally well, e.g., the role as a mother and the role as an employee. In the end, resulting overload and stress could lead to poorer maternal health [21]. As evidence clearly indicates the positive link between the mental health of mothers and their children [e.g., 22], maternal employment is, therefore, assumed to be associated with poorer child mental health. However, the exact opposite would be expected following the enhancement hypothesis. Here, a fixed amount of time and energy is denied. Instead, it is postulated that every new role adds sources of self-esteem and social support which in the end leads to better health [23, 24].

In conclusion, depending on the theory chosen [for a broader overview please refer to 19, 25] different hypotheses on the association of maternal employment and child mental health emerge. Besides that, as different as theoretical considerations may seem at first glance, they do have one thing in common. It is assumed that maternal employment is not directly linked to child mental health, but via *mediators*, e.g., maternal health or financial resources. In the end, positive and negative associations could even offset each other resulting in a missing link [18]. Furthermore, *moderators* also have to be considered as maternal employment could be differently associated with mental health among subgroups of children. Examples for moderators include children’s age or timing of maternal employment after birth, respectively. Especially early maternal return to work has been extensively researched and discussed, e.g., based on concepts of attachment and critical periods in children’s lives [see 25].

### Previous research on the association of maternal employment and child mental health

The extensive body of research on the association of maternal employment and child mental health has so far produced mixed findings leading to different conclusions [see 20, 25]. In recent years, various reviews emphasized that such an association, if any, may arise when early maternal employment is investigated. Especially employment within the first year postpartum has been shown to be adversely related to child mental health in terms of BP [26–28], whereas later employment could even be beneficial [26]. Concerning intensity, working very long hours was seen as a potential issue [26]. Further important variables include ethnicity [27] due to possible differences in, e.g., normativity of maternal employment and the home environment [see 18], maternal preferences [26], and working conditions [26], including working schedules [29] and income [26, 27].

A meta-analysis that also focused on early maternal employment, i.e., during child age 0–3 years, found somewhat different associations with BP. Here, children of employed mothers showed less internalizing behavior problems (IBP) than children of unemployed mothers. This finding extended to overall and externalizing behavior problems (EBP) in samples consisting predominantly of one-parent families. However, children of mothers working full-time exhibited more BP and EBP than children of unemployed mothers [25].

Evidence on the association between maternal employment and child mental health is mixed. However, it clearly emphasizes the importance of mediators and moderators as was already theoretically assumed. Furthermore, it is not only maternal employment per se that seems to matter. *Working characteristics*, i.e., amount, timing, and duration (how long the mother had been back at work after birth), have to be considered as well. Part-time rather than full-time employment, returning to work only after the first year postpartum, and employment stability could be especially beneficial for child mental health [e.g., 25, 26, 30]. However, due to limitations of previous research, no definite conclusions can yet be drawn. The results of the meta-analysis by Lucas-Thompson and colleagues [25] exemplify this. First, authors focused on overall measures of mental health, i.e., BP, EBP, and IBP. However, maternal employment could be differently associated with subordinate measures such as aggression or hyperactivity. Second, results were aggregated over a wide range of children’s age, i.e., including studies that measured mental health at some time during infancy, childhood, or adolescence. However, maternal employment may be differently linked to child mental health depending on child age. As younger children cannot take care of themselves, having a working mother may have stronger implications in infancy and early childhood. Third, included studies originated predominantly from the US, e.g., using data of the *National Longitudinal Survey of Youth* (NLSY79) that investigates children born by the end of the last century. In recent years, more investigations focusing on children born considerably later added to the literature, e.g., the *Early Childhood Longitudinal Study—Birth Cohort* (ECLS-B). As maternal workforce participation has increased in recent decades [e.g., 17] and thus became more normative, maternal employment could have different implications for children depending on their year of birth. Finally, maternal employment covers more than employment status per se or working amount (including comparisons of full-time and part-time employment). When also focusing on additional employment characteristics, e.g., duration or timing of return to work, more distinctive conclusions could emerge.

Hence, lack of associations between maternal employment and child mental health could be a result of aggregating different health measures that are either positively or negatively linked to employment. Furthermore, mixing results of studies that investigated different age groups could be an explanation. The associations found could also be biased by the same mechanisms. Moreover, it remains unclear whether results can be generalized to more recent findings on the association of maternal employment and child mental health including data generated by newer cohort studies. Beside maternal employment status, other employment characteristics may be important as well.

### Objectives and research questions

This systematic review with meta-analysis aimed at summarizing recent evidence on the association of maternal employment and child mental health to update findings and overcome shortcomings of previous research. In addition to focusing on maternal employment status and working amount, we also summarized results on employment duration and timing of return to work. Child mental health was operationalized in terms of overall measures, e.g., BP, and subordinate measures, e.g., aggression. Studies to be included had to investigate children of a specific age group, i.e., 0–7 years. Starting school marks an important developmental transition. Furthermore, as children get older, they can take better care of themselves. These circumstances could possibly alter the implications of maternal employment. Hence, such a narrow frame of children’s age was warranted.

## Methods

A comprehensive literature search was conducted to identify and summarize the results of recent observational studies examining the association of maternal employment and child mental health. The review’s study protocol had been pre-registered and is available online (PROSPERO-ID: CRD42018109227, please see the most recent version; https://www.crd.york.ac.uk/prospero/display_record.php?RecordID=109227).

### Search

Initially, three different databases (PsycINFO, PubMed, and Web of Science Core Collection) were searched for relevant literature from 2005 to 2020. The year 2005 was considered as starting point to include recent evidence only [for an analysis of research from 1960 to 2010 please refer to 25]. Evidence published in 2021 and 2022 was not included. In those years, studies often focused on COVID-19. As the pandemic impacted both maternal employment, e.g., by school closures [31], and child mental health [e.g., 32], we would have introduced bias. A comprehensive search string was used consisting of keywords describing the population, treatment and comparison conditions, and the outcomes of interest (for more details please refer to the Online Resource and see Supplementary Box S1). Additionally, reference lists of included studies, the previously published meta-analysis [25], and former reviews [26, 29, 33] were manually screened for further relevant literature. Dissertations and working papers were considered, if available, to cover gray literature.

### Eligibility criteria

To be eligible, studies had to focus on children 0–7 years of age. Studies were excluded if the sample (also) comprised children eight years or older. In terms of maternal employment, studies were included if children of working mothers were compared to children of non-working mothers. Further eligible comparisons included working more (e.g., full-time) vs. less (e.g., part-time) hours, longer vs. shorter (in terms of employment duration), or sooner vs. later after birth. In either case, maternal employment had to constitute the independent variable. Studies investigating family employment only, e.g., by inclusion of grandmothers or fathers, were excluded. If an appropriate control group was missing studies were excluded as well.

Child mental health could be operationalized by BP, e.g., EBP or IBP, or subordinate measures, e.g., aggression or anxiety. Prosocial behavior (PB) constituted an additional but positive outcome. The (non-) significance of the investigated association with maternal employment had to be clearly stated, i.e., in text form or by provided figures. Studies were excluded if the studies’ outcome referred to other areas than mental health, e.g., somatic conditions, cognitive development, or skill formation. Self-esteem and temperament were not considered eligible outcomes either.

Concerning study design, observational investigations published in English or German language were considered eligible. Intervention studies, reviews or meta-analyses, case reports, case series studies, commentaries, editorials, and expert opinions were excluded. To be eligible for the meta-analytic part, studies had to additionally report a measure of effect size or present information allowing for its calculation.

### Study selection

Eligibility of studies was examined by screening based on title/abstract first. Afterward, full-texts were retrieved and assessed for inclusion. In both cases, this was done by two reviewers (M.K., M.L.) independently. If consensus could not be reached, a third reviewer (S.G.-N.) was consulted; majority won.

### Data extraction and coding of study variables

Data were extracted by two reviewers (M.K., M.L.) using a standardized extraction form [34] modified based on the review’s inclusion criteria. Maternal employment was represented as a categorical variable if studies provided employment *status* (employed vs. unemployed, full-time vs. part-time employment) or results on *timing* of return to work. In contrast, maternal employment constituted a continuous variable if studies provided information on working *amount* (working hours) or employment *duration*. Four categories of child mental health outcomes were considered. BP referred to measures of behavior in general, e.g., measured by use of the total score of the *Strengths and Difficulties Questionnaire* [SDQ; 35]. EBP were assessed, e.g., by subscales of the *Child Behavior Checklist* [e.g., 36] and included aggression, hyperactivity/inattention, conduct problems, and disruptive behavior. IBP were assessed, e.g., by subscales of the SDQ and referred to emotional problems, anxious/depressed behavior, and withdrawn behavior/peer problems. PB was assessed, e.g., by the corresponding subscale of the SDQ and covered positive and cooperative behavior.

Potential moderators included study characteristics, i.e., data source (cohort study or country data originated from), design (cross-sectional, longitudinal), and whether presented results were adjusted for confounders (no, yes). Based on previous research and theoretical assumptions, ethnicity, child sex, child and maternal age, maternal marital status, education, and health were considered confounders. Studies’ methodological quality (low, moderate, or high risk of bias) was assessed by two reviewers (M.K., M.L.) and constituted a further potential moderator. We used an adapted risk of bias procedure based on Ijaz and colleagues [37]. Some aspects of the initial checklist were omitted as they were not considered relevant for our systematic review with meta-analysis. Examples include blinding (not feasible in observational studies) and reporting of hypotheses tested (association of maternal employment and child behavior did not constitute the research focus of every included study). The resulting checklist consists of five major domains essential for study quality (e.g., recruitment procedure and measurement of maternal employment and child behavior) and three minor domains (e.g., chronology and funding). For nearly every domain, risk of bias was considered low, high, or unclear. Study-level risk of bias was then rated as low (i.e., low risk in every major domain and at least two minor domains), moderate (i.e., low risk in at least four major domains and one minor domain), or high (i.e., low risk in less than four major domains or no minor domain). If consensus could not be reached a third reviewer (S.G.-N.) was consulted; majority won.

Sample characteristics considered as potential moderators included child age at outcome assessment (in years; if an age range was given the mean was used for analyses), child sex (girls vs. boys vs. mixed), and timing of maternal employment after birth (in years; if a range was given the mean was used for analyses). Ethnicity of mother and/or child was coded as majority White vs. majority Black vs. majority Hispanic (with majority referring to at least 75% of the sample) vs. mixed vs. not reported. Categorization of maternal marital status included majority coupled mothers vs. majority lone mothers (with majority again referring to at least 75% of the sample) vs. mixed vs. not reported.

### Syntheses of results and meta-analytic procedures

First, narrative syntheses of evidence for the various outcomes under investigation were conducted. If both unadjusted and adjusted analyses were carried out, only results of the adjusted analyses were considered. In case of various adjusted analyses, we focused on results of the fully-adjusted models.

Meta-analytic syntheses of evidence followed if at least two studies either provided directly corresponding, comparable effect sizes or data that allowed their calculation. The effect size used depended on the intervention’s measurement level. For categorical employment indices, the *Odds Ratio* (OR) was calculated. Maternal unemployment or part-time employment constituted the respective reference categories. In case of continuous employment indices, the correlation coefficient *r* was calculated. A positively signed value indicated that working more or longer was associated with a higher level of the respective outcome. Note that interpretation, therefore, differs for negatively labeled outcomes, e.g., BP (higher score is unfavorable), and positively labeled outcomes, e.g., PB (higher score is good). In case *OR* or *r* were not provided directly, they were calculated based on other available statistics. To include results based on *β*, we followed the approach of Peterson and Brown [38]. In general, adjusted results were preferred. However, if adjusted values did not allow for effect size calculation unadjusted values were used. Effect sizes were generated with the study as the unit of analysis. If a study provided data on independent subgroups, e.g., girls and boys, or various comparisons, e.g., employed full-time vs. not employed and employed part-time vs. not employed, the mean of these associations was used to form a single effect size, e.g., employed vs. not employed. In case a single study yielded multiple associations based on the same sample, e.g., when measuring the respective outcome twice, a combined effect size was used to prevent from violation of the assumption of independence. Summary effects were then computed by means of a random-effects model. For meta-analyses including five or less studies, results based on the fixed-effects model were considered, too (the estimation of between-study variance is less precise when the summary effect’s calculation is based on such a small number of studies [39]). Heterogeneity, referring to variation in results between studies, was evaluated using the *Q* statistic. If heterogeneity had to be assumed, moderator analyses followed. Unadjusted effects of each potential moderator variable were assessed and supplemented by analyses examining the joint contribution of important moderators, if possible. Level of significance was set at *p* < 0.05. Again, studies served as units of analysis. Only in case, a single study yielded multiple associations based on independent subgroups of sample-level moderators these subgroups served as units of analysis. If multiple associations of single studies still had to be combined (see above) covering different manifestations of study- or sample-level moderators, we handled this dependent on the moderator under investigation. For design, a third category (mixed) was introduced. Child age and timing of maternal employment were either set to the value on which most associations were based or to the respective mean value if majority was missing.

All analyses were carried out with *Comprehensive Meta-Analysis* [40]. For moderator analyses, the metaregression tool was used.

## Results

**Fig. 1:**
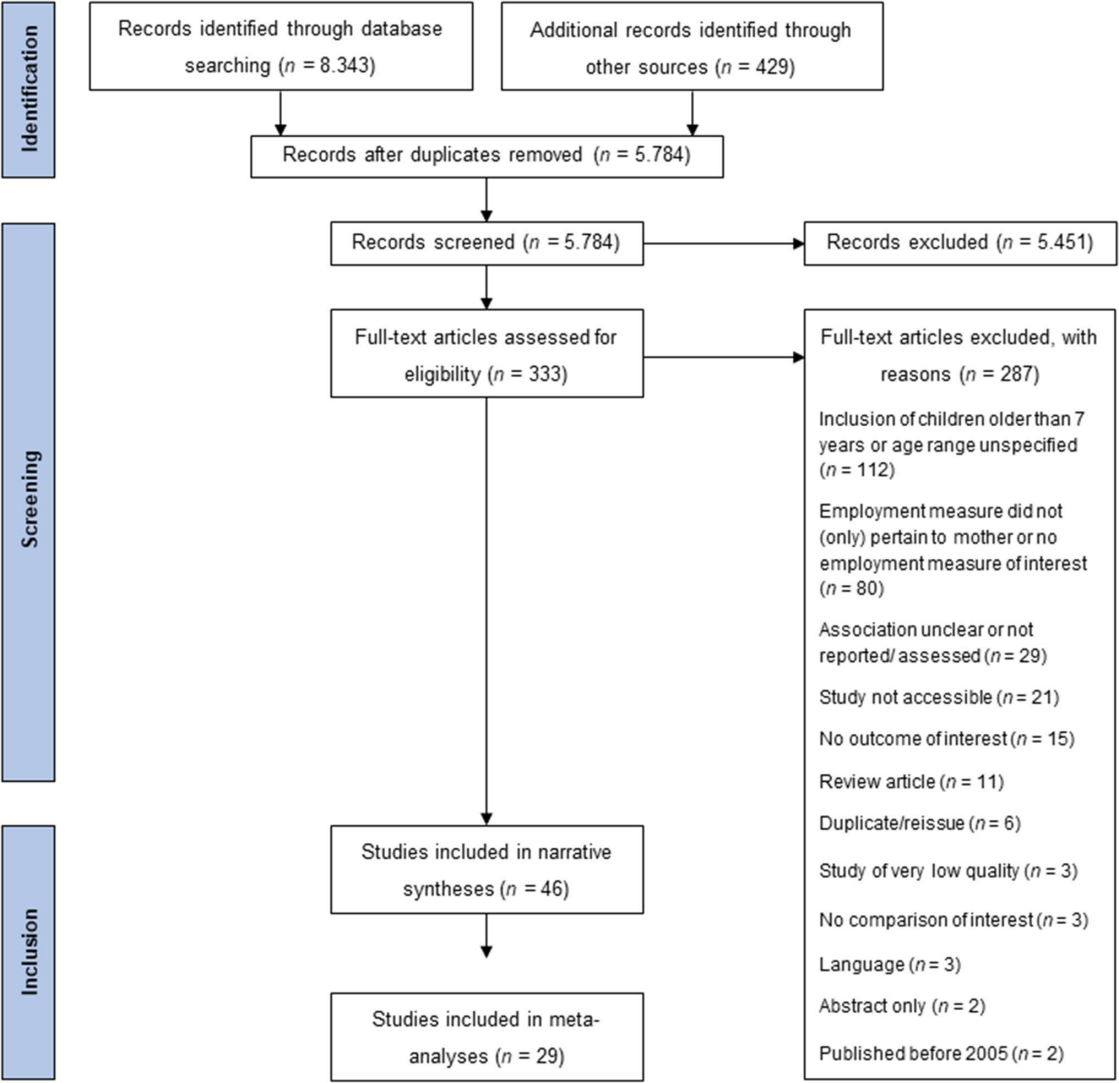
Flowchart

With duplicates removed, the search process yielded 5,784 results. These were initially screened for eligibility based on title and abstract, resulting in the retrieval of 333 full-texts. Figure 1 depicts the whole process in more detail. In total, 46 studies were considered in the narrative syntheses; 29 studies were included in the meta-analytic syntheses. Please refer to the Online Resource for a corresponding reference list.

Table 1 provides brief information on study- and sample-level characteristics, for more details please refer to the Online Resource (Supplementary Tables S1, S2, and S3; extensive information on results of included studies appear in Supplementary Tables S4, S5, S6, and S7). In total, 39 studies focused on maternal employment status or working amount, whereas eight studies investigated employment duration, and six studies included analyses on timing of return to work. Child mental health was operationalized by BP in 18 studies, by EBP in 27 studies, by IBP in 17 studies, and by PB in nine studies. Analytical sample sizes varied from *n* = 41 to *n* = 13,160. In 22 studies, the association of interest was investigated longitudinally and nearly every study provided adjusted results. Most research originated from the US and relied on data of large cohort studies. Unfortunately, risk of bias had to be considered high for a huge majority (see Supplementary Table S3). Turning to sample characteristics, children were ten months [41] to 7.5 years old [42, 43] at outcome assessment. Studies investigated the association in both sexes, with only few of them providing distinct estimates for girls and boys. Timing of maternal employment varied from the first year [e.g., 44] to 7 years postpartum [45]. Most studies focused on samples of mixed ethnicity investigating (majority) coupled mothers.

**Table 1:** Study characteristics

Characteristic	*k*	*%*
Publication source		
Journal article	37	80.4
Thesis	3	6.5
Working paper	2	4.3
Book chapter	1	2.2
Discussion paper	1	2.2
Monography	1	2.2
Report	1	2.2
Data source^a^		
US	27	58.7
FFCWS	7	
ECLS-B	6	
NLSY79	3	
NICHD-SECCYD	2	
CCDP	1	
IDP	1	
PHDCN	1	
Other	6	
United Kingdom	6	13.0
MCS	3	
ALSPAC	2	
GUS	1	
Australia	4	8.7
LSAC	3	
Other	1	
Denmark	2	4.3
DALSC	2	
Canada	1	2.2
NLSCY	1	
Germany	1	2.2
ENEPS	1	
Indonesia	1	2.2
Iran	1	2.2
Japan	1	2.2
South Korea	1	2.2
Turkey	1	2.2
Intervention		
Employment status (incl. working amount)	39	
Employment duration	8	
Return to work	6	
Outcome		
Behavior problems	18	
Externalizing behavior problems	27	
Aggression	7	
Hyperactivity/inattention	6	
Conduct problems	3	
Internalizing behavior problems	17	
Anxiety and depressive symptoms	7	
Other	2	
Prosocial behavior	9	
Design		
Longitudinal	22	47.8
Cross-sectional analyses only	24	52.2
Risk of bias		
Low	4	8.7
Moderate	11	23.9
High	31	67.4
Child age at outcome assessment (in years)		
0–1	1	
2	4	
3	13	
4	16	
5	12	
6	7	
7	3	
Timing of maternal employment		
0–12 months	22	
13–23 months	12	
2 years	12	
3 years	11	
4 years	17	
5 years	9	
6 years	1	
7 years	1	
Ethnicity^a^		
Mixed	18	39.1
(Majority) White	6	13.0
(Majority) Black	3	6.5
n/a	19	41.3
Marital status		
Mixed	14	30.4
(Majority) coupled	21	45.7
(Majority) single	5	10.9
n/a	6	13.0

*k* refers to the number of respective studies

*FFCWS* Fragile Families and Child Wellbeing Study, *ECLS-B* Early Childhood Longitudinal Study—Birth Cohort, *NLSY79* National Longitudinal Survey of Youth, *NICHD-SECCYD* National Institute of Child Health and Human Development Study of Early Child Care and Youth Development, *CCDP* National Impact Evaluation of the Comprehensive Child Development Program, *IDP* Interactional and Developmental Processes Study, *PHDCN* Project on Human Development in Chicago Neighborhoods, *MCS* Millennium Cohort Study, *ALSPAC* Avon Longitudinal Study of Parents and Children, *GUS* Growing Up in Scotland, *LSAC* Growing up in Australia, the Longitudinal Study of Australian Children, *DALSC* Danish Longitudinal Survey of Children, *NLSCY* National Longitudinal Survey of Children and Youth, *ENEPS* Erlangen-Nürnberger Entwicklungs- und Präventionsstudie (Erlangen-Nuremberg Development and Prevention Study), *n/a* not available/applicable

^a^Values do not add up to 100% due to rounding

In the following sections, we first present narrative syntheses of evidence for every outcome under investigation. Meta-analytic evidence follows, if applicable. The focus is on statistically significant results, with full details provided in Table 2. Moderator analyses are mentioned only when they could be performed (for more details please refer to Online Resource, Supplementary Table S8).

**Table 2:** Results of meta-analyses

	*k*	Random-effects model	Fixed-effects model	Heterogeneity
Employment status				
Overall BP	9	*OR* = 0.668 [0.432–1.032]	n/a	*Q *(8) = 373.067***
Overall EBP	7	*OR* = 1.031 [0.869–1.224]	n/a	*Q *(6) = 40.077***
Aggression	4	*OR* = 1.168 [0.705–1.935]	*OR* = 0.865 [0.726–1.032]	*Q *(3) = 15.251**
Hyperactivity/inattention	3	*OR* = 0.956 [0.780–1.172]	*OR* = 1.041 [0.970–1.117]	*Q *(2) = 12.437**
Conduct problems	2	*OR* = 1.180** [1.055–1.320]	*OR* = 1.168*** [1.086–1.257]	*Q *(1) = 2.147
Overall IBP	3	*OR* = 0.843 [0.692–1.027]	*OR* = 0.868*** [0.810–0.931]	*Q *(2) = 6.96*
Anxiety and depressive symptoms	3	*OR* = 0.664 [0.404–1.093]	*OR* = 0.804* [0.671–0.963]	*Q *(2) = 9.917**
PB	5	*OR* = 0.997 [0.785–1.266]	*OR* = 1.020 [0.973–1.068]	*Q *(4) = 94.522***
Full-time vs. part-time employment				
Overall BP	4	*OR* = 0.668 [0.432–1.032]	*OR* = 1.020 [0.941–1.106]	*Q *(3) = 22.918***
Overall EBP	3	*OR* = 1.271 [0.816–1.980]	*OR* = 1.119* [1.010–1.239]	*Q *(2) = 15.147**
Hyperactivity/inattention	2	*OR* = 3.003 [0.305–29.571]	*OR* = 2.677*** [2.222–3.226]	*Q *(1) = 149.022***
Overall IBP	2	*OR* = 1.068 [0.925–1.234]	*OR* = 1.068 [0.925–1.234]	*Q* (1) = 0.031
PB	2	*OR* = 0.979 [0.789–1.216]	*OR* = 1.027 [0.927–1.138]	*Q *(1) = 2.522
Employment duration				
Overall BP	2	*r* = − 0.072** [− 0.123 –− 0.020]	*r* = − 0.082*** [− 0.109–− 0.055]	*Q* (1) = 2.168
Overall EBP	3	*r* = 0.012 [− 0.071–0.095]	*r* = 0.038*** [0.018–0.058]	*Q *(2) = 28.105***
Overall IBP	2	*r* = − 0.075 [− 0.220–0.073]	*r* = − 0.072*** [− 0.103–− 0.040]	*Q *(1) = 21.764***
PB	2	*r* = 0.047 [− 0.054–0.147]	*r* = 0.023* [0.000–0.047]	*Q *(1) = 14.391***

*BP* behavior problems, *n/a* not available/applicable, *EBP* externalizing behavior problems, *IPB* internalizing behavior problems, *PB* prosocial behavior

*k* refers to the number of respective studies

Significant results appear in bold

Note that even in case heterogeneity had to be assumed moderator analyses could not always be conducted due to the small number of studies included (see Online Resource, Supplementary Table S8)

### Overall behavior problems

Comparing children of employed and unemployed mothers, 6 out of 13 studies [30, 41, 46–55] pointed to employment being associated with less BP [30, 46, 49, 54–56]. Subgroup analyses of Berger and colleagues [48] emphasized the importance of ethnicity. In Hispanic children, maternal employment was linked to higher levels of BP, whereas in Black children, it was associated with lower levels of BP.

Our corresponding meta-analysis including nine studies [30, 46, 48–54] indicated no association of maternal employment status and BP based on the random-effects model. Moderator analyses pointed to data source and ethnicity being of importance. Taking data source as dummy for country, in British data (under comparison to US data) being employed was linked to lower BP (*b* = -0.817, *P* < 0.001). In samples of mixed ethnicity being employed was linked to more BP when compared to (majority) White samples (*b* = 0.927, *P* < 0.01). Due to the small number of studies, joint effects of data source and ethnicity could not be assessed.

Focusing on full-time vs. part-time employment, results of six available studies [30, 50, 51, 57–59] did not yield any associations with BP. This was replicated in our corresponding meta-analysis including four studies [30, 50, 51, 58]. Moderator analyses pointed to adjustment for confounders and timing of maternal employment being of importance. Full-time employment was linked to more BP in adjusted data (*b* = 0.454, *P* < 0.05) and when maternal employment was measured later in children’s lives (*b* = 0.121, *P* < 0.05). In an analysis including both characteristics, i.e., adjustment for confounders and timing of maternal employment, none of the single coefficients remained significant.

**Fig. 2:**
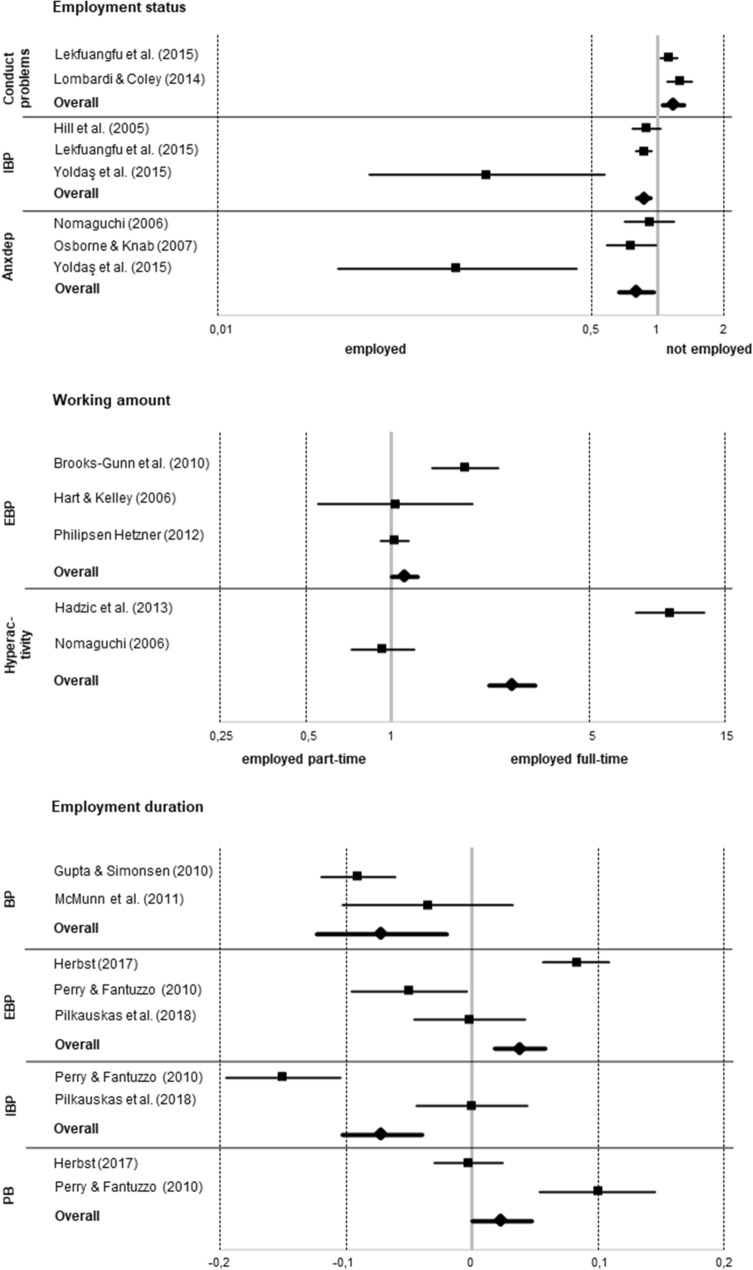
Forest-plots on meta-analyses yielding summary effects; *IBP* internalizing behavior problems, *anxdep* anxiety and depressive symptoms, *EBP* externalizing behavior problems, *BP* behavior problems, *PB* prosocial behavior

Four more studies [30, 42, 43, 49] focused on employment duration. In analyses of Kiernan and Mensah [49] and McMunn and colleagues [30], both using British data, single results indicated longer employment being linked to lower BP. Our corresponding meta-analysis including two studies [30, 43] indeed indicated an association of maternal employment duration and BP based on both the random-effects model (*r* = − 0.072, *P* < 0.01) and the fixed-effects model (*r* = − 0.082, *P* < 0.001). Longer employment was associated with less BP (see Fig. 2).

Only a single study [51] included analyses on timing of return to work. Here, no interpretable results emerged. Overall, we thus found employment duration to be linked to child BP. Evidence of an association between employment status and BP was only found in a narrative synthesis.

### Externalizing behavior problems

*Overall externalizing behavior problems.* Comparing children of employed and unemployed mothers, five out of 11 studies [6, 18, 50, 60–67] yielded significant but mixed results. Employment was linked to either less [6, 65] or more EBP [6, 62, 63, 67], the latter especially in cases of full-time employment [62, 63]. Our corresponding meta-analysis including seven studies [6, 18, 50, 62–65] indicated no association of maternal employment and EBP based on the random-effects model. Moderator analyses followed but none of the variables under investigation was relevant.

Focusing on full-time vs. part-time employment, four studies [44, 65, 68, 69] did not yield any associations, whereas results of Hill and colleagues [62] as well as Haas [70] pointed to full-time employment or more maternal working hours being linked to more EBP. Analyses of Brooks-Gunn and colleagues [18] again emphasized the importance of ethnicity. In White children, limited evidence pointed to full-time employment being associated with higher levels of EBP, whereas this was shown for part-time employment in Black children instead. In our corresponding meta-analysis including three studies [18, 50, 69] working full-time indeed was associated with more EBP under the fixed-effects model (*OR* = 1.119, *P* < 0.05; see Fig. 2). Moderator analyses followed but none of the variables under investigation was relevant.

Further three studies [19, 61, 71] focused on employment duration with a single study finding more months of employment (within the same job) being associated with less EBP [19]. Our corresponding meta-analysis based on all three studies indeed indicated an association of maternal employment duration and EBP based on the fixed-effects model, albeit pointing to longer employment being associated with more EBP instead (*r* = 0.038, *P* < 0.001; see Fig. 2).

Four studies [18, 44, 62, 63] included further analyses on timing of return to work and several of them pointed to early return, i.e., within the first year postpartum, being associated with more EBP [18, 44, 63]. As these analyses were not comparable to each other, no corresponding meta-analysis followed. Overall, we thus found full-time employment and employment duration to be linked to child EBP. Evidence of an association between timing of return to work and EBP was found in a narrative synthesis.

*Aggression*. Comparing children of employed and unemployed mothers, three out of five studies [72–76] pointed to employment being associated with more aggression [72, 74, 75]. However, the opposite was found as well [74, 75], especially when mothers received higher levels of social support. Our corresponding meta-analysis including four studies [72–75] did not yield a summary effect. Moderator analyses followed, but none of the variables under investigation turned out to be of importance.

A further analysis of Pilarz [77] found part-time employment to be linked to less aggression. Whitaker and colleagues [78] focused on the association between employment duration and aggression. Here, no results were obtained. Overall, we thus found no association of maternal employment and child aggression.

*Hyperactivity/inattention*. Four studies [41, 63, 74, 79] compared children of employed and unemployed mothers. Lekfuangfu and colleagues [63] found maternal full-time employment to be linked to more hyperactivity/inattention, while analyses of Nomaguchi [74] yielded limited evidence of part-time (part-year) employment being associated with less hyperactivity. Here, in our corresponding meta-analysis including three studies [63, 74, 79], no summary effect emerged.

Focusing on full-time vs. part-time employment, Hadzic and colleagues [80] found 35–40 maternal weekly working hours to be directly linked to more hyperactivity/inattention when compared to 16–34 working hours. Taking maternal parenting practices as mediating variables into account, further indirect effects emerged linking 1–15 and more than 40 working hours to less hyperactivity/inattention. Indeed, our corresponding meta-analysis including two studies [74, 80] indicated an association of maternal employment and hyperactivity/inattention based on the fixed-effects model (*OR* = 2.677, *P* < 0.001). Here, working full-time was linked to more hyperactivity/inattention (see Fig. 2).

A further analysis on employment duration did not yield any associations [78]. The same holds true for studies focusing on timing of return to work [63, 79]. As these analyses were not comparable to each other, no meta-analysis was conducted. Overall, we thus found full-time employment to be linked to child hyperactivity/inattention.

*Conduct problems.* Comparing children of employed and unemployed mothers, one out of two studies [63, 79] found full-time employment to be linked to more conduct problems [63]. Our corresponding meta-analysis including both studies indeed yielded a summary effect based on the random-effects model (*OR* = 1.180, *P* < 0.01) and the fixed-effects model (*OR* = 1.168, *P* < 0.001). Employment was linked to more conduct problems (see Fig. 2).

Focusing on full-time vs. part-time employment, Hadzic and colleagues [80] found 1–15 and more than 40 weekly working hours to be indirectly linked to less conduct problems when compared to 16–34 working hours. In terms of return to work, analyses yielded mixed results. Lekfuangfu and colleagues [63] found limited evidence of early return, i.e., between seven and 12 months postpartum, to be associated with more conduct problems. However, results of Lombardi and Coley [79] pointed to an association with later return instead, i.e., between 9 and 24 months postpartum, especially when working part-time and with increasing household income. However, this link was no longer present when further mediating variables were included in the model. As these analyses were not comparable to each other, no meta-analysis followed. Overall, we thus found employment status to be linked to child conduct problems.

### Internalizing behavior problems

*Overall internalizing behavior problems.* Of six studies comparing children of employed and unemployed mothers [60, 62, 63, 66, 67, 81], only Im and Vanderweele [67] found results linking employment to more IBP (especially in cases of absent or low paternal involvement). In contrast, when paternal involvement was high, employment was associated with less IBP. In analyses of Yoldaş and colleagues [81] children of working mothers exhibited less IBP. Our corresponding meta-analysis including three studies [62, 63, 81] yielded a summary effect under the fixed-effects model (*OR* = 0.868, *P* < 0.001), indicating that children of employed mothers exhibited less IBP (see Fig. 2).

Focusing on full-time vs. part-time employment, in four studies no interpretable results emerged [62, 68, 69, 77] and the same was true for the corresponding meta-analysis including two studies [62, 69]. Two further studies focused on employment duration yielding at least limited evidence of more IBP in children of mothers being unemployed for a longer time [19, 71]. Our corresponding meta-analysis including these studies was conducted with the fixed-effects model yielding a summary effect (*r* = − 0.072, *P* < 0.001). Being employed for a longer time was linked to less IBP (see Fig. 2).

In terms of return to work analyses of Lekfuangfu and colleagues [63] included limited evidence that returning within 13–18 postpartum months may be associated with less IBP when compared to not having returned to work by 18 months postpartum. Hill and colleagues [62], however, did not find any associations. As these analyses were not comparable to each other, no meta-analysis followed. Overall, we thus found employment status and duration to be linked to child IBP.

*Anxiety and depressive symptoms.* Of six studies comparing children of employed and unemployed mothers [45, 61, 73, 74, 76, 81], only two studies [74, 81] included limited evidence linking employment to less anxiety or anxious/depressed behavior. Our corresponding meta-analysis based on three studies [73, 74, 81] yielded a summary effect under the fixed-effects model (*OR* = 0.804, *P* < 0.05), indicating that children of employed mothers exhibited less anxious/depressed behavior (see Fig. 2).

Two further studies focused on employment duration [61, 78] with only Whitaker and colleagues [78] finding that children of mothers employed for a shorter time are more likely to exhibit problematic anxious/depressed behavior. No meta-analysis was conducted as the respective outcomes were not considered similar enough to be combined. Overall, we thus found employment status to be linked to child anxious/depressed behavior.

*Other internalizing symptoms*. Lekfuangfu and colleagues [63] investigated emotional and peer problems but did not find any differences between children of employed and unemployed mothers. However, having returned to work by 7–12 months postpartum was linked to less emotional and peer problems when compared to not having returned to work by 18 months postpartum. Osborne and Knab [73] focused on withdrawn behavior also not finding any differences between children of employed and unemployed mothers. Overall, limited evidence of an association between the timing of return to work and child emotional and peer problems was found in a narrative synthesis.

### Prosocial behavior

Comparing children of employed and unemployed mothers, four out of seven studies [41, 50, 61, 63, 74, 79, 80] yielded limited evidence for more PB in children of employed mothers [41, 50, 74, 80]. However, in our corresponding meta-analysis including five studies [50, 61, 63, 74, 79], no summary effect emerged. Moderator analyses pointed to marital status as an important variable. In samples with mixed marital status being employed was linked to less PB compared to (majority) coupled samples (*b* = − 0.179, *P* < 0.05).

Focusing on full-time vs. part-time employment, Hadzic and colleagues [80] found indirect links of 1–15 maternal weekly working hours and more PB as well as of more than 40 h and less PB when compared to 16–34 weekly working hours. In analyses of Gassman-Pines [68] more working hours during nights were linked to less positive behavior (especially on weekends). Our corresponding meta-analysis including two studies [50, 74] did not yield a summary effect.

Two further studies [61, 71] focused on employment duration with limited evidence pointing to less PB in children of mothers being unemployed for a longer time [71]. Our corresponding meta-analysis including these studies yielded a summary effect under the fixed-effects model (*r* = 0.023, *P* < 0.05), linking longer employment duration to more PB (see Fig. 2).

In terms of return to work, analyses included limited evidence of early return, i.e., within the first year postpartum, to be associated with less PB [63, 79]. As these analyses were not comparable to each other, no meta-analysis followed. Overall, we thus found employment duration to be linked to child PB. Evidence of an association between timing of return to work and PB was found in a narrative synthesis.

## Discussion

This systematic review with meta-analysis aimed at summarizing recent evidence on the association between maternal employment and mental health of children 0–7 years of age. We focused on employment status, working amount, employment duration, and timing of return to work and ran analyses for every single indicator of child mental health found, if possible. Generally, meta-analytic evidence pointed to only few associations between maternal employment and child mental health depending on the operationalization of maternal employment and the outcome studied. Moderator analyses yielded even fewer results but were often hampered by small numbers of studies included.

*Employment status.* Single studies provided evidence of employment being linked not only to somewhat less BP and more PB, but also to more aggression. However, meta-analytic syntheses did not confirm these results. Instead, maternal employment turned out to be linked to *more* conduct problems, i.e., externalizing behavior, but *less* IBP and anxious/depressed behavior in children. Analyses often included children 5–7 years of age with maternal employment pertaining to the first years postpartum. Therefore, these contradictory results may not be explained by differences in child age or timing of maternal employment. We may focus on child care instead. Children of working mothers are generally more likely to be placed in (nonmaternal) child care [82] and spend more time in such care arrangements [18]. This in turn has been found to be associated with child BP especially in terms of more EBP [83–85] but possibly less IBP [83]. Time spent with other children may be a potential mechanism and could both be detrimental and beneficial [85], e.g., by being exposed to more anti- or prosocial peer behavior when being placed in child care for more hours [86].

Especially analyses on BP or externalizing behavior, i.e., overall EBP, aggression, and hyperactivity/inattention, were characterized by inconclusive evidence and marked heterogeneity. Turning to moderators, data source needs to be considered and indicates regional or even cultural differences in the association between maternal employment and child mental health. In US data, maternal employment was associated with more BP. In a British sample being employed was linked to less BP instead. Both countries share quite comparable maternal employment rates with mothers working being the norm [87]. However, compared to the US, employment conditions in various European countries seem more supportive of mothers by providing more generous maternal leave entitlements [88]. Longer maternal leave has been shown to benefit maternal mental health [e.g., 89] which in turn is associated with better child mental health [22]. Furthermore, beside employment per se, as our meta-analytic evidence revealed, the actual amount of work seems to matter: Full-time employment was associated with more EBP and more hyperactivity/inattention. In the US, mothers mostly work full-time, whereas part-time work is more frequent in some European countries, e.g., the United Kingdom, Germany, or Switzerland [87]. Regional differences in the association between maternal employment and child mental health are further underlined by ethnicity being a moderator. Among others, factors such as socio-economic status, job characteristics, and parenting may be possible explanations. For instance, Black mothers seem to exhibit more harsh parenting behavior than White mothers do [90], which in turn has shown to be associated with problem behavior in children [e.g., 91, 92]. Employment and care by others then could compensate for that.

In terms of PB, also no summary effect emerged. However, maternal marital status was found to be a moderator. In samples including a significant number of lone mothers, employment was linked to less PB. Hence, this expands on findings from a former meta-analysis [25] that underscored the importance of family structure for the association of employment and problem behavior: Family structure could be essential for the link to positive behavior as well. Possible mechanisms may include family income which has been shown to be linked to child mental health problems [93] and is often substantially lower in one-parent families.

*Full-time vs. part-time employment.* Limited evidence was found for full-time employment to be linked to more EBP and more hyperactivity/inattention in children (which for the latter has to be interpreted with caution as results were mainly driven by large associations found in a single study). Both full-time and part-time working mothers benefit from the advantages of being employed, e.g., greater financial resources [18] and better maternal (mental) health [94, 95]. These in turn are closely linked to child mental health [22, 93]. However, the role of time in child care (with the potential need of full-time working mothers to place their children in child care for even more hours) has already been discussed. Further studies found maternal sensitivity to be higher among part-time working mothers [maybe attributable to selection effects; 18, 96] which in turn is negatively associated with EBP [97]. Once more, at least in US data, ethnicity may also play a role. Brooks-Gunn and colleagues [18] found that full-time employment was associated with more EBP in White children, whereas in Black children part-time employment was associated with more EBP. Unfortunately, due to the small number of studies included, we were not able to include ethnicity in moderator analyses focusing on EBP.

None of our further meta-analyses yielded summary effects. This may pertain to differing definitions of full-time and part-time employment across studies. In general, heterogeneity played a smaller role compared to analyses on employment status: Moderators were only identified in relation to BP. Here, adjustment for confounders was relevant. In general, differences in child mental health dependent on maternal employment often diminish when controlling for selection into employment, e.g., by inclusion of maternal education as confounder. However, we found full-time employment to be linked to BP when focusing on *adjusted* results. Therefore, other factors than the confounders we considered (examples include maternal age, marital status, or mental health) have to explain this interesting finding. We already discussed the potential roles of child care and parenting in this context.

*Employment duration.* Single analyses underscored an association of employment stability and child mental health. Longer employment duration turned out to be linked to less BP, less IBP, and more PB. Again, financial security or income may reflect underlying mechanisms. Employment stability also seems to be of importance for employees’ mental health [98] with the clear association of maternal and child mental health already having been discussed. Job churning or job loss may also be linked to less stability in child care arrangements, the latter of which has been shown to be important for child mental health [82]. Despite that, longer employment duration was also associated with more EBP albeit included studies pointed to a missing or even negative link. Heterogeneity was given but due to the small number of studies included, again no moderator analyses followed. Once more, child care could mark a possible mechanism. Children of mothers employed for a longer time may cumulatively spend more time in child care which in turn has been associated with more EBP [84]. However, before actually resolving conflicting evidence, more research on the association of maternal employment duration and EBP is highly warranted.

*Return to work.* Only few studies focused on timing of maternal employment rendering any meta-analysis impossible. However, narrative syntheses indicated early maternal return to work, i.e., within the first 12 months postpartum, to be associated with worse child mental health, i.e., more EBP and less PB. For the latter, this may seem inconsistent with conclusions drawn for employment duration as analyses of Herbst [61], which focused on employment duration within the first 9 months postpartum, failed to show any cumulative negative link between employment and PB. However, employment duration is looking at *how stably* a mother is employed, even early after birth, whereas in return-to-work analyses, *when* a mother best resumes employment is investigated. Therefore, these analyses are not fully comparable to each other. However, one could assume that potential detrimental effects of returning to work early may be offset if this employment is at least stable. Unfortunately, as both analyses relied on only two studies the evidence is too scarce to draw any final conclusions.

Early return to work being linked to worse child mental health is consistent with analyses on employment status. Studies that found maternal employment to be linked to more EBP often investigated employment within the first year postpartum. Furthermore, studies indicating a positive link of maternal employment and PB mainly focused on employment *later* in children’s life.

Indeed, based on prior investigations, early return has been considered an issue. Policies on family leave, including both maternal and paternal leave, are closely linked to timing of return to work. The positive association of maternal leave and maternal (mental) health [e.g., 89] has already been discussed and in turn, maternal and child mental health are closely linked to each other [22]. Besides that, many countries are now offering paternal leave as well, but its take-up by fathers is low [99], although it has already been shown to be detrimental for maternal mental health if the partner does not take any paternal leave [89]. Hence, negative associations of mothers’ early return to work and child mental health may be buffered in case paternal leave is utilized instead. However, this needs further research. Shorter durations of breastfeeding due to early return to work [100] constitute another potential mechanism. Limited evidence indicates a positive association of breastfeeding and child mental health [101, 102]. Again, child care may pose an explanation as well. When mothers return to work earlier children may have to start child care at younger ages. Especially attendance of child care within the first year postpartum has been shown to be negatively associated with child behavior [103].

*Summary.* Our findings suggest that whether maternal employment is associated with child mental health strongly depends on the operationalization of maternal employment, the outcome under investigation, and study/sample characteristics. Especially part-time employment, longer employment duration, and return to work only after the first year postpartum may be beneficial for child mental health. Moderator analyses pointed in particular to regional differences regarding the association between maternal employment and child mental health.

### Strengths and limitations

Previous research on the association between maternal employment and child mental health was limited by focusing solely on broader measures of maternal employment, e.g., status or working amount, and child mental health, e.g., EBP or IBP. Moreover, evidence was often obtained by mixing results on different age groups, i.e., infants, children, and adolescents. These approaches may have resulted in mistakenly missing associations between maternal employment and child mental health. Strengths of this systematic review with meta-analysis therefore lie in its additional focus on employment duration and return-to-work analyses and in running analyses for every single indicator of child mental health found, if possible. Moreover, we only included studies investigating children 0–7 years of age. By doing so, we avoided mixing results that are barely comparable. Also, we concentrated on very recent evidence on the association of the variables of interest. Special attention was further given to the investigation of moderators. Additional strengths include the systematic literature search as well as the standardized procedures of data extraction and assessment of study quality.

However, some limitations need to be considered as well. First, despite the clear focus on young children, included studies were still characterized by high levels of heterogeneity. Unfortunately, we were not able to fully explain this heterogeneity, because often only a few studies could be included in the meta-analyses. Even when moderator analyses were run, we may have missed further important variables. Second, especially analyses on employment duration and subordinate measures of externalizing and internalizing behavior were often complicated by the fact that there were few studies that had looked at them. As a compromise, we also considered summary effects based on the fixed-effects model. However, these analyses can be considered exploratory. Third, models comparing full-time and part-time employment may further be hampered by the fact that single studies did not use consistent definitions for these categories. Studies also considered different sets of control variables and relied on different analytical techniques. Fourth, generalizability of findings is further hampered by most included studies originating from the Western world (US in particular). Even here, moderator analyses pointed to regional differences in the association between maternal employment and child mental health. Differences in the normativity of full-time or part-time work and employment conditions mark possible explanations. Furthermore, due to the inclusion of cross-sectional investigations, we can only speak of associations rather than *effects* so far. Especially analyses that find benefits of children whose mothers work need to consider reverse causality. Mental health could precede maternal employment, i.e., having a healthier child enables her/his mother to work. However, with analyses focusing on timing of return to work, we were able to invalidate this explanation, as the evidence included often pointed to *later* return to be associated with better mental health. Besides that, we also included longitudinal investigations that pointed to working being linked to better child mental health. A final limitation concerns our exclusive focus on maternal employment. Hence, no conclusions on the association of paternal employment or leave-taking and child mental health can be drawn.

### Outlook

Practical implications pertain to the endorsement of maternal employment per se and employment stability in particular, especially after the first year postpartum as return-to-work analyses often demonstrated that working early after birth is associated with worse child mental health. Here, more generous maternal leave policies are warranted granting paid leave covering the first year postpartum. As initial results already pointed to the importance of *family* leave, e.g., by demonstrating links to maternal mental health [89] and results of Liu and colleagues [104] showing a direct association between shared parental caregiving and child behavior, further recommendations pertain to an expanded offer of paternal leave and the promotion of its actual use. In terms of full-time employment being linked to worse child mental health, buffering factors warrant consideration. Examples include support of maternal sensitivity, e.g., through specific interventions and enhancement of child care quality.

Implications for future research pertain to study methodology, populations, interventions, and outcomes under investigation. At study level, more investigations outside of the US are warranted utilizing, e.g., adequate analytical strategies, a comparable set of control variables, and consistent definitions of full-time and part-time employment. This is highly warranted especially in analyses on subordinate measures of externalizing and internalizing behavior, e.g., conduct problems or anxiety and depressive symptoms, before drawing firm conclusions. This also accounts for analyses on employment duration and return to work with special focus on any interdependence among these two variables. Implications of the COVID-19 pandemic for the association between maternal employment and child mental health may be of further interest for future research. Here, already altered gender role attitudes on maternal employment [105] or employment conditions, e.g., working from home [106], may pose potential mechanisms. In terms of systematic investigations, further research should focus on associations in children aged 8 years and above to examine whether maternal employment is differently linked to child mental health in infancy, childhood, or adolescence. Here, special focus could be given to the investigation of potential logged associations [26] and further subgroup analyses shedding light on additional important moderators. The systematic investigation of any association between child mental health and other employment factors than those investigated here, e.g., number of jobs held and job quality, falls under this heading. Furthermore, systematic investigations focusing on an aggregated measure of *parental* employment instead of maternal conditions only could be promising. In this way, more knowledge is generated about when and how children benefit from (maternal) employment, so that further implications for practice can be derived.

## Conclusion

This systematic review with meta-analysis aimed at summarizing recent evidence on the association between maternal employment and child mental health. Evidence suggests that whether maternal employment is linked to child mental health strongly depends on the operationalization of maternal employment, the outcome being studied, and study/sample characteristics, e.g., data source. Children of part-time working mothers may fare better than children whose mothers work full-time. This also applies to children of mothers employed for a longer time. Early return to work, i.e., within the first year postpartum, was often associated with poorer child mental health. However, as was already pointed out by Greenstein [20], maternal employment will not be uniformly detrimental or beneficial for children. Knowledge on when and how negative or positive associations emerge is rather crucial. As our moderator analyses were often limited by too few studies, more research on underlying mechanisms, e.g., leave entitlements, child care, and maternal sensitivity, is clearly needed, especially for the development of effective prevention and intervention strategies.

## Supplementary Information

Below is the link to the electronic supplementary material.

Supplementary file1 (DOCX 141 KB)

## Data Availability

The data underlying this article will be shared on reasonable request to the corresponding author.
