# Supplementary material for: Association between maternal employment and the child´s mental health: a systematic review with meta-analysis
Source: Eur Child Adolesc Psychiatry. 2023 Feb 13;33(9):3025–42. doi: 10.1007/s00787-023-02164-1 (PMC9924214; doi:10.1007/s00787-023-02164-1)
Supplement: Supplementary file 1 — Supplementary file1 (DOCX 141 KB) [file 787_2023_2164_MOESM1_ESM.docx]

**Supplementary materials**

**References of studies included in systematic review** (studies marked with * were included in meta-analyses as well)

*Berger L, Brooks-Gunn J, Paxson C, Waldfogel J (2008) First-year maternal employment and child outcomes: differences across racial and ethnic groups. Child Youth Serv Rev 30:365-387. https://doi.org/10.1016/j.childyouth.2007.10.010

*Berger LM, Hill J, Waldfogel J (2005) Maternity leave, early maternal employment and child health and development in the US. Econ J 115:F29-F47. https://doi.org/10.1111/j.0013-0133.2005.00971.x

*Brooks-Gunn J, Han W-J, Waldfogel J (2010) First-year maternal employment and child deveIopment in the first seven years. Monogr Soc Res Child Dev 75:1-148

Cabrera N, Hofferth SL, Hancock G (2014) Family structure, maternal employment, and change in children's externalizing problem behaviour: Differences by age and self-regulation. Eur J Dev Psychol 11:136-158. https://doi.org/10.1080/17405629.2013.873716

*Conway A, Han W-J, Brooks-Gunn J, Waldfogel J (2017) First-year maternal employment and adolescent externalizing behavior. J Child Fam Stud 26:2237-2251. https://doi.org/10.1007/s10826-017-0730-8

Deding M, Lausten M, Rosenstjerne Andersen A (2007) Starting school: the effect of early childhood factors on child well-being. Working paper, The Danish National Institute of Social Research

Dunifon R, Kalil A, Crosby DA, Su JH (2013) Mothers' night work and children's behavior problems. Dev Psychol 49:1874-1885. https://doi.org/10.1037/a0031241

Fiori F (2020) Maternal employment and the well-being of children living with a lone mother in Scotland. Demographic Res 43:1685-1738. https://doi.org/10.4054/DemRes.2020.43.57

Gassman‐Pines A (2011) Low‐income mothers' nighttime and weekend work: daily associations with child behavior, mother‐child interactions, and mood. Fam Relat 60:15-29. https://doi.org/10.1111/j.1741-3729.2010.00630.x

*Goodman G, Aber JL (2010) Predictors of representational aggression in preschool children of low-income urban African American adolescent mothers. Infant Ment Health J 31:33-57. https://doi.org/10.1002/imhj.20241

*Gupta ND, Simonsen M (2010) Non-cognitive child outcomes and universal high quality child care. IZA Discussion Papers, No. 3188, Institute for the Study of Labor (IZA)

Haas KB (2014) Mothers' nonstandard work schedules and young children's school readiness. Dissertation, Loyola University Chicago

*Hadzic R, Magee CA, Robinson L (2013) Parental employment and child behaviors: do parenting practices underlie these relationships? Int J Behav Dev 37:332-339. https://doi.org/10.1177/0165025413477274

Hall LA, Rayens MK, Peden AR (2008) Maternal factors associated with child behavior. J Nurs Scholarsh 40:124-130. https://doi.org/10.1111/j.1547-5069.2008.00216.x

*Hart MS, Kelley ML (2006) Fathers' and mothers' work and family issues as related to internalizing and externalizing behavior of children attending day care. J Fam Issues 27:252-270. https://doi.org/10.1177/0192513X05280992

*Herbst CM (2017) Are parental welfare work requirements good for disadvantaged children? Evidence from age-of-youngest-child exemptions. J Policy Anal Manage 36:327-357. https://doi.org/10.1002/pam.21971

*Hill JL, Waldfogel J, Brooks-Gunn J, Han WJ (2005) Maternal employment and child development: a fresh look using newer methods. Dev Psychol 41:833-850. https://doi.org/10.1037/0012-1649.41.6.833

Im Y, Vanderweele TJ (2018) Role of first‐year maternal employment and paternal involvement in behavioral and cognitive development of young children. Infant Ment Health J 39:449-465. https://doi.org/10.1002/imhj.21716

*Jackson AP, Choi J-K, Bentler PM (2009) Parenting efficacy and the early school adjustment of poor and near-poor Black children. J Fam Issues 30:1339-1355. https://doi.org/10.1177/0192513X09334603

Jackson AP, Choi J-K, Franke TM (2009) Poor single mothers with young children: mastery, relations with nonresident fathers, and child outcomes. Soc Work Res 33:95-106. https://doi.org/10.1093/swr/33.2.95

*Jaursch S, Losel F (2011) Mütterliche Berufstätigkeit und kindliches Sozialverhalten [Mothers' employment and children's social behavior]. Kindh Entwickl 20:164-172. https://doi.org/10.1026/0942-5403/a000052

*Kiernan KE, Mensah FK (2009) Poverty, maternal depression, family status and children's cognitive and behavioural development in early childhood: a longitudinal study. J Soc Policy 38:569-588. https://doi.org/10.1017/S0047279409003250

Law C, Hope S, Petticrew M, Roberts H, Whitehead M (2014) In what circumstances can parental employment improve child health. Final report, Public Health Research Consortium

*Le Zhang M, Boyd A, Cheung SY, Sharland E, Scourfield J (2020) Social work contact in a UK cohort study: under-reporting, predictors of contact and the emotional and behavioural problems of children. Child Youth Serv Rev 115:105071. https://doi.org/10.1016/j.childyouth.2020.105071

*Lekfuangfu WN, Powdthavee N, Clark AE, Ward G (2015) Early maternal employment and non-cognitive outcomes in early childhood and adolescence: evidence from British birth cohort data. CEP Discussion Paper No 1380, Centre for Economic Performance, London School of Economics and Political Science

*Lombardi CM, Coley RL (2014) Early maternal employment and children’s school readiness in contemporary families. Dev Psychol 50:2071-2084. https://doi.org/10.1037/a0037106

*McMunn A, Kelly Y, Cable N, Bartley M (2012) Maternal employment and child socio-emotional behaviour in the UK: longitudinal evidence from the UK Millennium Cohort Study. J Epidemiol Community Health 66:e19. https://doi.org/10.1136/jech.2010.109553

*Meysamie A, Ghalehtaki R, Ghazanfari A, Daneshvar-Fard M, Mohammadi MR (2013) Prevalence and associated factors of physical, verbal and relational aggression among Iranian preschoolers. Iran J Psychiatry 8:138-144

*Miller-Lewis LR, Baghurst PA, Sawyer MG, Prior MR, Clark JJ, Arney FM, et al. (2006) Early childhood externalising behaviour problems: child, parenting, and family-related predictors over time. J Abnorm Child Psychol 34:891-906. https://doi.org/10.1007/s10802-006-9071-6

*Nomaguchi KM (2006) Maternal employment, nonparental care, mother-child interactions, and child outcomes during preschool years. J Marriage Fam 68:1341-1369. https://doi.org/10.1111/j.1741-3737.2006.00332.x

*Osborne C, Knab J (2007) Work, welfare, and young children's health and behavior in the Fragile Families and Child Wellbeing Study. Child Youth Serv Rev 29:762-781. https://doi.org/10.1016/j.childyouth.2006.12.005

Park MH, Yim HW, Park S, Lee C, Lee CU, Hong SC, et al. (2015) School refusal behavior in South Korean first graders: a prospective observational community-based study. Psychiatry Res 227:160-165. https://doi.org/10.1016/j.psychres.2015.04.011

Pekkurnaz D (2015) Dynamic analysis of maternal employment, child care, quality and early childhood development. Dissertation, University of North Carolina

*Perry MA, Fantuzzo JW (2010) A multivariate investigation of maternal risks and their relationship to low-income, preschool children's competencies. Appl Dev Sci 14:1-17. https://doi.org/10.1080/10888690903510281

*Perry-Jenkins M, Laws HB, Sayer A, Newkirk K (2020) Parents' work and children's development: a longitudinal investigation of working-class families. J Fam Psychol 34:257-268. https://doi.org/10.1037/fam0000580

*Philipsen Hetzner NM (2012) First year parental employment and child developmental outcomes at two and four years of age. Dissertation, Columbia University

Pilarz AR (2021) Mothers' work schedule inflexibility and children's behavior problems. J Fam Issues 42:1258-1284. https://doi.org/10.1177/0192513x20940761

*Pilkauskas NV, Brooks-Gunn J, Waldfogel J (2018) Maternal employment stability in early childhood: links with child behavior and cognitive skills. Dev Psychol 54:410-427. https://doi.org/10.1037/dev0000438

Rosenbaum E, Morett CR (2009) The effect of parents’ joint work schedules on infants’ behavior over the first two years of life: evidence from the ECLSB. Matern Child Health J 13:732-744. https://doi.org/10.1007/s10995-009-0488-8

*Strazdins L, Obrien LV, Lucas N, Rodgers B (2013) Combining work and family: rewards or risks for children's mental health? Soc Sci Med 87:99-107. https://doi.org/10.1016/j.socscimed.2013.03.030

Strazdins L, Shipley M, Clements M, Obrien LV, Broom DH (2010) Job quality and inequality: parents' jobs and children's emotional and behavioural difficulties. Soc Sci Med 70:2052-2060. https://doi.org/10.1016/j.socscimed.2010.02.041

*Sugawara M (2005) Maternal employment and child development in Japan: a twelve-year longitudinal study. In: Shwalb DW, Nakazawa J, Shwalb BJ (eds) Applied developmental psychology: theory, practice, and research from Japan. IAP Information Age Publishing, Charlotte, pp 225-240

*Sundaur AH, Rustina Y (2019) The correlation between caregivers' characteristics and emotional development of pre-school children in Depok, Indonesia. Compr Child Adolesc Nurs 42:245-251. https://doi.org/10.1080/24694193.2019.1594456

Turney K (2012) Pathways of disadvantage: explaining the relationship between maternal depression and children's problem behaviors. Soc Sci Res 41:1546-1564. https://doi.org/10.1016/j.ssresearch.2012.06.003

Whitaker RC, Orzol SM, Kahn RS (2006) Maternal mental health, substance use, and domestic violence in the year after delivery and subsequent behavior problems in children at age 3 years. Arch Gen Psychiatry 63:551-560. https://doi.org/10.1001/archpsyc.63.5.551

*Yoldaş T, Yoldaş TC, Beyazal M, Sayıcı U, Örün UA (2020) Relationship between non-cardiac chest pain and internalizing problems in pre-school aged children. Cardiol Young 30:1261-1265. https://doi.org/10.1017/S1047951120001948

**Supplementary Box S1: Search string**

The listed search string was used for PsycINFO. Slight modifications were necessary for searching PubMed and Web of Science Core Collection.

ab((maternal OR mother*) AND (employ* OR unemploy* OR work* OR “non work” OR nonworking OR job* OR “part time” OR “full time” OR “part-time” OR “full-time” OR “maternity leave” OR labor OR labour) AND (child* OR infancy OR infant* OR boy* OR girl* OR toddler* OR childhood OR preschool OR pre-school) AND (“behavior problem*” OR “behaviour problem*” OR “behavioural outcome*” OR “behavioral outcome*” OR “child behavior*” OR “child behaviour*” OR external* OR internal* OR “emotional disorder*” OR “emotional problem*” OR “emotional development” OR socioemotional OR “childhood emotional disorder*” OR hyperactiv* OR hyperkinetic OR “child* anxiety” OR “childhood anxiety” OR “separation anxiety” OR “social anxiety” OR “conduct disorder*” OR “conduct problem*” OR aggress* OR withdraw* OR defiant OR defiance OR “disturbance of activity and attention” OR “attachment disorder*” OR “social functioning” OR “selective mutism” OR “phobic anxiety disorder in childhood” OR stuttering OR “tic disorder*” OR “well being” OR “well-being”))

| **Supplementary Table S1.** Study- and sample-level characteristics. | | | | | | | | | |
| --- | --- | --- | --- | --- | --- | --- | --- | --- | --- |
| **Study** | **Type** | **Data source (country), children's birth year(s)** | **Design** | **Sample size^a^** | **Ethnicity^b^** | **Child age^c^** (*M* (*SD*)) **and sex** | **Maternal age** (*M* (*SD*)) | **Maternal marital status** | **Socioeconomic status** |
| Berger et al. (2005) | Journal Article | NLSY79 (US), 1988–1996 | longitudinal | 574–769 | n/a | 4 years, n/a | 23–38 years | n/a | 100% employed at some point within three months before birth |
| Berger et al. (2008) | Journal Article | FFCWS (US), 1998–2000 | longitudinal | 1,328–1,483 | 59.7% Black, 20.2% White, 20.1% Hispanic | 37.7 (2.6) months, 51.4% male | 24.6 (5.8) years^d^ | 20.6% married^d^, further 37.4% cohabitating^d^ | 31.3% with less than high-school education^d^, predominantly poor or of low-income |
| Brooks-Gunn et al. (2010) | Monography | NICHD-SECCYD (US), 1991 | longitudinal | 49–925 | 88.8% White^k^, 11.2% Black^k^ | 3 years and 4.5 years, 49.0–51.9% male^l^ (White children), 40.4–57.7% male^l^ (Black children) | 29.1 (5.2) – 29.5 (5.0) years^d,l^ (White children), 23.4 (5.4) – 26.3 (5.1) years^d,l^ (Black children) | 90.3–97.6% married^d,l^ (White children), 51.1–71.2% married^d,l^ (Black children) | 14.1–15.2 as mean years of education^d,l^ (White children), 12.7–13.7 as mean years of education^d,l^ (Black children) |
| Cabrera et al. (2014) | Journal Article | NLSY79 (US), 1984 | cross-sectional | 4,967 | n/a | 4 years and 6 years, 51.0% male | n/a | 83% living in two-parent household (either with biological or step-father)^m^ | 13.0 as mean years of education^m^ |
| Conway et al. (2017) | Journal Article | NICHD-SECCYD (US), 1991 | longitudinal | 922 | 100% White | 3 years and 4.5 years, 52% male | 29.3 (5.2) years | 91% married^d^ | 14.5 as mean years of education |

| **Study** | **Type** | **Data source (country), children's birth year(s)** | **Design** | **Sample size^a^** | **Ethnicity^b^** | **Child age^c^** (*M* (*SD*)) **and sex** | **Maternal age** (*M* (*SD*)) | **Maternal marital status** | **Socioeconomic status** |
| --- | --- | --- | --- | --- | --- | --- | --- | --- | --- |
| Deding et al. (2007) | Working Paper | DALSC (Denmark), 1995 | longitudinal | 4,651 | n/a | 7.5 years, 51.9% male | 29.3 (4.6; girls) years^d^ and 29.5 (4.5; boys) years^d^ | 9% (girls) and 8% (boys) living with single mother^f^ | 13.4 as mean years of education^g^, 28% living in poor financial circumstances as of self-report^f^ |
| Dunifon et al. (2013) | Journal Article | FFCWS (US), 1998–2000 | cross-sectional | 2,367 (2,367–4,734 ob-servations^r^) | 51.0% Black, 24.0% Hispanic, 22.0% White, 3.0% Other | 4.0 (1.1) years^r^, 52.0% male | 29.2 (6.1) years^r^ | 39.0% lone mothers^r^ | 22.0% on welfare during last year^r^; 26.0% with less than high-school education, 13% with college degree or higher^r^ |
| Fiori (2020) | Journal Article | GUS, 2004–2005 or 2010–2011 | longitudinal | 918 | n/a | 5 years, 51.2% male | 15.4% below 20 years^d^ | 100% lone mothers at child age 3 years | 54.0% of low educational level^d^, 25.3% of households in bad financial situation at child age 3 years |
| Gassman-Pines (2011) | Journal Article | large north-eastern City (US), n/a | cross-sectional | 61 (724 ob-servations) | 65.6% Black, 23.0% Hispanic, 6.6% White, 1.6% Asian | 51.2 (7.3) months, 45.0% male | 30.6 (8.3) years | 47.9% single and not cohabitating, further 35.1% single but cohabitating | 100% employed; 100% of low income, 12.6 as mean years of education (85.3% with high school diploma) |
| Goodman & Aber (2010) | Journal Article | IDP (US), n/a | cross-sectional | 93 | 100% Black | 3.9 (0.6) years (range 3–5), 44.1% male | 21.3 (1.1) years, 100% adoles-cent mothers | 1.1% married | 100% low-income mothers |

| **Study** | **Type** | **Data source (country), children's birth year(s)** | **Design** | **Sample size^a^** | **Ethnicity^b^** | **Child age^c^** (*M* (*SD*)) **and sex** | **Maternal age** (*M* (*SD*)) | **Maternal marital status** | **Socioeconomic status** |
| --- | --- | --- | --- | --- | --- | --- | --- | --- | --- |
| Gupta & Simonsen (2007) | Working Paper | DALSC (Denmark), 1995 | longitudinal | 4,343 | n/a | 7.5 years | 27.84 (4.9) – 28.37 (4.6) depending on mode of care | depending on mode of care, 2–6% living with single mother |  |
| Haas (2013) | Thesis | ECLS-B (US), 2001 | cross-sectional | 2,879–3,480 | 60.9% White, 18.9% Hispanic, 14.9% Black, 2.6% Asian or Pacific Islander, 2.6% Other | approximately 4 years, 50.7% male | n/a | 67.1% married | 100% employed; 22.3% of low income, 7.7% with less than high school diploma, 13.3% with graduate degree |
| Hadzic et al. (2013) | Journal Article | LSAC (Australia), 2003–2004 | longitudinal | 2,271 | n/a | 6–7 years, 51.3% male | n/a | 100% partnered | 20.0% with below or at least high school education, 43.0 with university degree |
| Hall et al. (2008) | Journal Article | single mothers from Kentucky, at baseline of RCT (US), 1994–2000 | cross-sectional | 205 | approximately half White, half Black | 3.3 (1.3) years (range 2–6), 53% male | 27.0 (5.8) years | 100% single | at or below 185% of poverty level, >80% with at least high school education, >50% with at least some college education |
| Hart & Kelley (2006) | Journal Article | families from Virginia, n/a | cross-sectional | 132 | 71.2% White, 15.2% Black, 6.8% Hispanic, 5.3% multicultural, 1.5% Pacific Islander | 39.6 (11.6) months (range 1.5–4 years), 61.4% male | 32.3 (6.2) years | 100% living with partner (81.8% married) | 100% employed; 21% lower class, 68% middle class, 11% upper class; 9.1% with high school education only, 5.3% with graduate degree |

| **Study** | **Type** | **Data source (country), children's birth year(s)** | **Design** | **Sample size^a^** | **Ethnicity^b^** | **Child age^c^** (*M* (*SD*)) **and sex** | **Maternal age** (*M* (*SD*)) | **Maternal marital status** | **Socioeconomic status** |
| --- | --- | --- | --- | --- | --- | --- | --- | --- | --- |
| Herbst (2017) | Journal Article | ECLS-B (US), 2001 | longitudinal | 5,334–5,932 | 18.4% Black | 60 months, 51.7% male | 26.5 (6.0) years^d^ | 57.3% married^d^ | welfare-eligible sample (mothers not married and/or have less than college degree); 25.4% high school dropout^d^; 32.6% of household income below Federal Poverty Level, 66.6% participated in WIC program |
| Hill et al. (2005) | Journal Article | NLSY79 (US), 1982–1993 | longitudinal | 290–4,297 | 52% White, 28% Black, 20% Hispanic | 5–6 years, 51.0% male | 25.4 (4.1) years^d^ | 66% married^d^ | 26% households in poverty; 31% with less than high school education, 11% with college education^d^ |
| Im & Vanderweele (2018) | Journal Article | PHDCN (US), approx. 1994–1997 | longitudinal | 411 | 45.6–57.6% Hispanic, 25.4–27.8% Black, 12.3–24.7% White, 1.9–4.7% Other^x^ | 0.4 (0.2) years^w^, 51.1–55.6% male^w,x^ | 26.1 (6.9) – 26.9 (6.6) years^w,x^ | 25.5–29.2% single mothers^w,x^ | 9.0–15.3% below high school education, majority of low income^w,x^ |
| Jackson, Choi, & Bentler (2009)^e^ | Journal Article | families from Pittsburgh (US), n/a | cross-sectional, longitudinal | 100 | 100% Black | 3 years at first outcome assessment, 5 years at second outcome assessment; n/a | 25 years at first outcome assessment, 27 years at second outcome assessment | 100% single | 12.6 (SD = 1.2) as mean years of education; poor or near-poor (current or former welfare recipients) |
| **Study** | **Type** | **Data source (country), children's birth year(s)** | **Design** | **Sample size^a^** | **Ethnicity^b^** | **Child age^c^** (*M* (*SD*)) **and sex** | **Maternal age** (*M* (*SD*)) | **Maternal marital status** | **Socioeconomic status** |
| Jackson, Choi, & Franke (2009)^e^ | Journal Article | families from Pittsburgh (US), n/a | longitudinal | 86 | 100% Black | 5 years; n/a | 27 years^c^ | 100% single | 12.6 (SD = 1.2) as mean years of education; poor or near-poor (current or former welfare recipients) |
| Jaursch & Lösel (2011) | Journal Article | ENEPS (Germany), n/a | cross-sectional | 660 | n/a | 56.4 (9.3) months, 49.8% male | 34.5 (4.3) years^c^ | 87% married or living with partner^c^ | 13% low, 84% middle, 3% high^c^ |
| Kiernan & Mensah (2009) | Journal Article | MCS (United Kingdom), 2000–2002 | cross-sectional | 9,471 | 90.6% White, 3.4% Pakistani/ Bangladeshi, 2.2% Black, 1.8% Indian, 0.8% Mixed, 1.2% Other^i^ | 3 years, 50.8% male^i^ | 14.8% <20, 77.9% 20–34, and 7.2% >34 years old at first birth (in 42.7% index child was firstborn) | 14.1% single mothers^i^ | 10.2% without any qualifications^i^; 16.0% living in poverty at both 9 months and 3 years postpartum, further 16.1% living in poverty at either 9 months or 3 years postpartum |
| Law et al. (2014) | Report | MCS (United Kingdom), 2000–2002 | cross-sectional | 11,007–13,160 | n/a | 3, 5, and 7 years, n/a | n/a | n/a | n/a |
| Le Zhang et al. (2020) | Journal Article | ALSPAC (England), 1991–1992 | longitudinal | 7,951 | n/a | 42 months, 51.4% male | 97.9% older than 20 years^d^ | 5.4% single mothers | n/a |

| **Study** | **Type** | **Data source (country), children's birth year(s)** | **Design** | **Sample size^a^** | **Ethnicity^b^** | **Child age^c^** (*M* (*SD*)) **and sex** | **Maternal age** (*M* (*SD*)) | **Maternal marital status** | **Socioeconomic status** |
| --- | --- | --- | --- | --- | --- | --- | --- | --- | --- |
| Lekfuangfu et al. (2015) | Discussion Paper | ALSPAC (England), 1991–1992 | longitudinal | 1,698–8,857 | 93%–97% White^n^ | 47 and 81 months, 50.2% male | 28.3 (4.9) – 29.8 (4.7)^n^ | 3%–9% lone mothers^d,n^ | 37%–60% with education of at least A-level (16%–40% with university degree)^n^; 10–12% experienced major financial problem^n,o^ |
| Lombardi & Coley (2014) | Journal Article | ECLS-B (US), 2001 | cross-sectional, longitudinal | approx. 10,100 | 53.5% White, 25.5% Hispanic, 13.7% African American, 7.2% Other | 75.1 (9.2) months, 51.1% male | 28.2 (6.4) years | 23.9% never married | 15.7% received welfare at some study wave, 19.6% with less than high school education, 24.3% with bachelor´s or graduate degree |
| McMunn et al. (2011) | Journal Article | MCS (United Kingdom), 2000–2002 | cross-sectional, longitudinal^h^ | 9,504 | 100% White | 5 years, 52.9% male | n/a | 25.1% single^c^ | 8.7% without any educational qualifications^c^, 14.7% in poorest category of household income^c^ |
| Meysamie et al. (2013) | Journal Article | families in Tehran (Iran), 2002–2005 | cross-sectional | 1,403 | n/a | 3–6 years, 51.8% male^s^ | 11.4% below 18 or over 35 years old^s^ | n/a | 7.7% with education of at least 11 years, further 7.2% with education of more than 16 years^s^ |

| **Study** | **Type** | **Data source (country), children's birth year(s)** | **Design** | **Sample size^a^** | **Ethnicity^b^** | **Child age^c^** (*M* (*SD*)) **and sex** | **Maternal age** (*M* (*SD*)) | **Maternal marital status** | **Socioeconomic status** |
| --- | --- | --- | --- | --- | --- | --- | --- | --- | --- |
| Miller-Lewis et al. (2006) | Journal Article | families from the Adelaide metropolitan area (Australia), n/a | longitudinal | 332 | n/a | 6 years, 51.0% male | n/a | 15% living with single parent^m^ | 33% living in families that receive pension/ benefit; 52% with education at or below 12 years^m^ |
| Nomaguchi (2006) | Journal Article | NLSCY (Canada), 1994–1995 | cross-sectional, longitudinal | 1,099–1,178 | n/a | 2 years (29.8 (3.1) months) and 4 years, 51.1% male | 29.2 (4.9) years^t^ | 13.4% single at child age 2 years, 17.3% single at child age 4 years | 15.7% with less than high school education, 18.4% with university degree^t^ |
| Osborne & Knab (2007) | Journal Article | FFCWS (US), 1998–2000 | cross-sectional | 497–779 | 69.8% Black, 17.1% Hispanic | 35.8 (2.5) months, 53.6% male | 23.1 (5.1) years^d^ | 42.1% cohabitating^d^ | 100% (current or former) welfare recipients; 45.1% with less than high school, 19.2% with any college education^d^ |
| Park et al. (2015) | Journal Article | families from Seoul (South Korea), n/a | cross-sectional | 277 | n/a | 6–7 years, 52.3% male | n/a | 100% partnered | study was conducted in one of highest SES regions of South Korea; 79.1% college graduates |
| Pekkurnaz (2014) | Thesis | ECLS-B (US), 2001 | longitudinal | 21,150–21,450 ob-servations | 60.1% White, 20.6% Hispanic, 19.3% Black^p^ | 10.5 (1.9) – 61.4 (2.7) months (various waves combined)^v^, 51.2% male^p^ | 28.3 (6.4) years^p^ | 65.8% married^p^ | 26.5% with university degree^p^ |

| **Study** | **Type** | **Data source (country), children's birth year(s)** | **Design** | **Sample size^a^** | **Ethnicity^b^** | **Child age^c^** (*M* (*SD*)) **and sex** | **Maternal age** (*M* (*SD*)) | **Maternal marital status** | **Socioeconomic status** |
| --- | --- | --- | --- | --- | --- | --- | --- | --- | --- |
| Perry & Fantuzzo (2010) | Journal Article | national impact evaluation of CCDP (US), n/a | cross-sectional | 1,816 | 42.0% Black, 30.0% Hispanic, 28.0% White | 4 years, 50% male | 24.0 (5.7) years^d^ | n/a | 100% low-income; 10^th^ grade as mean of education^d^ |
| Perry-Jenkins et al. (2020) | Journal Article | working-class couples (US), approx. 1999–2001 | longitudinal | 120 | 92.2% White, 1.5% African American, 1.0% Hispanic | 6 years, 42.5% male | 27.8 years^y^ | 100% partnered | 100% employed before birth, 16.0% with high school diploma or General Education Diploma as highest education |
| Philipsen Hetzner (2011) | Thesis | ECLS-B (US), 2001 | longitudinal | 1,500–9,000 | 45.7% White, 17.8% Hispanic, 16.1% Black, 13.0% Asian, 7.1% Other | 24.5 (1.3) and 53.0 (4.2) months, 51.1% male | 11.3% younger than 20 years | 65.1% married and further 14.1% cohabitating, 20.0% single^p^ | 41.1% used WIC during pregnancy; 19.1% with less than high school education and 26.4% with college degree |
| Pilarz (2021; published online in 2020) | Journal Article | FFCWS (US), 1998–2000 | cross-sectional | 1,451 | 52.1% Black, 22.8% Hispanic 22.3% White, 2.8% Other | 61.1 (2.4) months, 50.9% male | 25.2 (6.0) years^d^ | 41.8% single^c^ | 100% employed^c^, 57% with incomes below 200% of FPL^q^, 25.5% with less than high school education^q^ |

| **Study** | **Type** | **Data source (country), children's birth year(s)** | **Design** | **Sample size^a^** | **Ethnicity^b^** | **Child age^c^** (*M* (*SD*)) **and sex** | **Maternal age** (*M* (*SD*)) | **Maternal marital status** | **Socioeconomic status** |
| --- | --- | --- | --- | --- | --- | --- | --- | --- | --- |
| Pilkauskas et al. (2017) | Journal Article | FFCWS (US), 1998–2000 | cross-sectional | 2,011 | 52.0% Black, 25.0% Hispanic, 20.0% White, 3.0% Other | 4.7 (0.5) years, 52.0% male | 24.9 (6.0) years | 21.0% married, further 37.0% cohabitating (42.0% single)^d^, over survey waves 31.0% are stably married or cohabitating, 14% were stably single | 33.0% were in poverty at 3 or 4 (out of 4) survey waves; 39.0% with less than high school education, 10.0% with college degree |
| Rosenbaum & Morett (2009) | Journal Article | ECLS-B (US), 2001 | longitudinal | 1,650 | 73–76% White, 14–17% Hispanic, 7% Black, 3–4% Asian | 24.2–24.3 months, 52–53% male | 30.8–32.1 years | 100% living in two-parent families^p^, 89–90% married | 100% employed^p;^ 4% in lowest and 25–34% in highest quintile |
| Strazdins et al. (2010) | Journal Article | LSAC (Australia), 1999–2000 | cross-sectional | 2,373 | n/a | 4–5 years, 50.2% male | 35.2 (0.1) years | 10.3% living with single mothers | 100% employed; 9.7% living with mothers of very low income; 14.8 (0.1) as mean years of education |
| Strazdins et al. (2013)^j^ | Journal Article | LSAC (Australia), 1999–2000 | cross-sectional | 2,809 | n/a | 4–5 years, 50.0% male | 35.0 (0.1) years | 10.5% living with single mothers | 14.2% low, 54.7% mid, and 31.1% high; 100% employed; 10.0% living with mothers of very low income; 14.7 (0.1) as mean years of education |
|  |  |  |  |  |  |  |  |  |  |

| **Study** | **Type** | **Data source (country), children's birth year(s)** | **Design** | **Sample size^a^** | **Ethnicity^b^** | **Child age^c^** (*M* (*SD*)) **and sex** | **Maternal age** (*M* (*SD*)) | **Maternal marital status** | **Socioeconomic status** |
| --- | --- | --- | --- | --- | --- | --- | --- | --- | --- |
| Sugawara (2005) | Book Chapter | mother-child-dyads from Kawasaki (Japan), 1984–1986 | longitudinal | 386 | n/a | 5 years old, n/a | n/a | >97.0% two-parent families | n/a |
| Sundaur & Rustina (2019) | Journal Article | families from Depok (Indonesia) | cross-sectional | 206 | n/a | 2% 3 years, 9% 4 years, 41% 5 years, 48% 6 years; 51.5% male | n/a (median age of 34 years) | n/a | 2.9% with low education |
| Turney (2012) | Journal Article | FFCWS (US), 1998–2000 | cross-sectional | 2,655 | 52.5% Black, 23.3% Hispanic, 21.5% White, 2.7% Other | 64.2 (2.9) months, 51.9% male | 25.0 (6.0)^q^ | 13.0% single^q^ | 60.6% Medicaid birth, 14.5% with material hardship^c^; 31.8% with less than high school education, 36.5% with post-secondary education^q^ |
| Whitaker et al. (2006) | Journal Article | FFCWS (US), 1998–2000 | longitudinal | 2,756 | 50.5% Black, 23.2% Hispanic, 23.1% White, 3.2% Other | 3 years, n/a | 18.2% <20 years, 59.3% 20–29 years, 22.5% at least 30 years old^d^ | 39.5% single^d^ | 43.3% below 1 in income-to-poverty ratio^u^; 31.8% with less than high school education, 11.0% at least college graduation^d^ |
|  |  |  |  |  |  |  |  |  |  |

| **Study** | **Type** | **Data source (country), children's birth year(s)** | **Design** | **Sample size^a^** | **Ethnicity^b^** | **Child age^c^** (*M* (*SD*)) **and sex** | **Maternal age** (*M* (*SD*)) | **Maternal marital status** | **Socioeconomic status** |
| --- | --- | --- | --- | --- | --- | --- | --- | --- | --- |
| Yoldaş et al. (2020) | Journal Article | patients of hospital in Ankara (Turkey) | cross-sectional | 41 | n/a | 2–5 years, 40% male | 24–45 years | n/a | 30% below high school education |

NLSY79 = National Longitudinal Survey of Youth; FFCWS = Fragile Families and Child Wellbeing Study; n/a = not available/applicable; NICHD-SECCYD = National Institute of Child Health and Human Development Study of Early Child Care and Youth Development; DALSC = Danish Longitudinal Survey of Children; IDP = Interactional and Developmental Processes Study; ECLS-B = Early Childhood Longitudinal Study – Birth Cohort; LSAC = *Growing up in Australia*, the Longitudinal Study of Australian Children; RCT = randomized controlled trial; WIC = Special Supplemental Nutrition Program for Women, Infants, and Children; PHDCN = Project on Human Development in Chicago Neighborhoods; ENEPS = Erlangen-Nürnberger Entwicklungs- und Präventionsstudie (Erlangen-Nuremberg Development and Prevention Study); MCS = Millennium Cohort Study; ALSPAC = Avon Longitudinal Study of Parents and Children; NLSCY = National Longitudinal Survey of Children and Youth; SES = socioeconomic status; CCDP = Comprehensive Child Development Program; FPL = Federal Poverty Line.

^a^Refers to analytical sample. ^b^Refers either to mothers or children. ^c^At outcome assessment. ^d^At childbirth. ^e^Same dataset as Jackson, Choi, & Bentler (2009). ^f^ At child age 3.5 years. ^g^At child age 6 months. ^h^Unadjusted analyses only. ^I^Refers to larger sample (not analytical sample). ^j^Same dataset as Strazdins et al. (2010). ^k^Refers to sample size *n* = 1.013. ^l^Values provided for groups based on maternal employment status by 12 months postpartum (working full-time, working part-time, not working) not for sample as a whole. ^m^At child age 4 years. ^n^Descriptives provided for 4 different groups, i.e., returned to work part-time or full-time between months 0–18, returned to work between months 19–33, or returned to work later than 33 months postpartum, not for sample as a whole. ^o^Before birth. ^p^At child age 9 months. ^q^Shortly after childbirth. ^r^Based on pooled data across 3 and 5 years postpartum. ^s^Based on: Meysamie A, Fard MD, Mohammadi M-R. Prevalence of attention-deficit/hyperactivity disorder symptoms in preschool-aged Iranian children. *Iran J Pediatr* 2011;**21**:467-72. ^t^In year of birth. ^u^At 12 months postpartum. ^v^Not reported for final wave. ^w^At baseline in first year postpartum. ^x^Values provided for groups based on maternal employment status in first year postpartum. ^y^During pregnancy.

| **Supplementary Table S2.** Intervention, control, and outcomes under investigation. | | | | | | | | | | | |
| --- | --- | --- | --- | --- | --- | --- | --- | --- | --- | --- | --- |
| **Study** | **Intervention** | **Control** | **Outcome** | **Outcome operationalization** | **Statistical method(s)** | **Adjusted for** | | | | | |
|  |  |  |  |  |  | sex^a^ | age^b^ | marital status^c^ | educa-tion^c^ | health^c^ | ethnicity^d^ |
| Berger et al. (2005) | 1. returning to work within 12 weeks after birth  2. returning to work full-time within 12 weeks after birth | 1. not returning to work within 12 weeks after birth  2. returning to work part-time within 12 weeks after birth | EBP at 4 years | EBP subscale, e.g., covering antisocial behavior, hyperactivity, and peer problems, of BPI (Q), mother-report, sum score | OLS regression with and without propensity score matching | yes | yes | yes | yes | no | yes |
| Berger et al. (2008) | working within first year postpartum (at some point) | not working within first year postpartum | BP at 36 months | combination of anxious/depressed, withdrawn, and aggressive behavior by use of subscales of CBCL 1½–5 (Q), mother-report | OLS regression with and without propensity score matching | yes | yes | yes | yes | yes | yes |
| Brooks-Gunn et al. (2010) | 1. working full-time at 12 months postpartum^l^ knknnknkn  2. working part-time at 12 months postpartum | 1. working part-time/not working at 12 months postpartum  2. working full-time or not working at 12 months postpartum | EBP at 3 years and 4.5 years | EBP subscale, i.e., covering attention problems and aggressive behavior, of CBCL (Q), mother-report and caregiver-report, sum score and use of cut-off to define level of clinically meaningful problem behavior | OLS regression, SEM^m^ | yes | yes | yes | yes | yes | yes^i^ |

| **Study** | **Intervention** | **Control** | **Outcome** | **Outcome operationalization** | **Statistical method(s)** | **Adjusted for** | | | | | |
| --- | --- | --- | --- | --- | --- | --- | --- | --- | --- | --- | --- |
|  |  |  |  |  |  | sex^a^ | age^b^ | marital status^c^ | educa-tion^c^ | health^c^ | ethnicity^d^ |
| Brooks-Gunn et al. (2010) | 3. full-time return to work by 3, 6, or 9 months postpartum  4. part-time return to work by 3, 6, or 9 months postpartum  5. employed at 15, 24, or 36 months, ever employed between months 42 and 54 | 3. part-time or no return to work by 3, 6, or 9 months postpartum  4. full-time or no return to work by 3, 6, or 9 months postpartum  5. not employed at 15, 24, or 36 months, not employed between months 42 and 54 |  |  |  |  |  |  |  |  |  |
| Cabrera et al. (2014) | average weekly working hours at child age 4 years (since last interview at child age 2 years) | n/a | EBP at 4 years and change in EBP from 4 to 6 years | EBP subscale, e.g., covering antisocial behavior, hyperactivity, and peer problems, of BPI (Q), parent-report, sum score | SEM (latent difference score model) | yes | no^j^ | yes | yes | no | no |
| Conway et al. (2017) | 1. working full-time at any assessment within first year postpartum  2. working part-time at every assessment within first year postpartum | not working at every assessment within first year postpartum | EBP at 3 years and 4.5 years | EBP subscale, i.e., covering aggression and destructive behavior, of CBCL (Q), mother- and caregiver-report, sum score (averaged at 3 years, individually considered at 4.5 years)^o^ | SEM^n^ | yes | yes | yes | yes | yes | yes^f^ |

| **Study** | **Intervention** | **Control** | **Outcome** | **Outcome operationalization** | **Statistical method(s)** | **Adjusted for** | | | | | |
| --- | --- | --- | --- | --- | --- | --- | --- | --- | --- | --- | --- |
|  |  |  |  |  |  | sex^a^ | age^b^ | marital status^c^ | educa-tion^c^ | health^c^ | ethnicity^d^ |
| Deding et al. (2007) | 1. months employed within first year postpartum  2. months employed within years 1–3 postpartum | n/a | BP at 7.5 years | combination of hyperactivity/inatten-tion, emotional, conduct, and peer problems by use of SDQ (Q), parent-report, creation of three groups of children based on sum score (normal, borderline, abnormal) | ordered probit model | yes | yes | yes | yes | yes | no |
| Dunifon et al. (2013) | 1. 1–19, 20–34, 35–44, or 45+ weekly working hours (measured at 3 and 5 years postpartum) | 1. not working (measured at 3 and 5 years postpartum) | 1. aggressive behavior and anxious/depressed behavior at 3 years and 5 years  2. change in aggressive behavior and anxious/depressed behavior between 3 and 5 years postpartum | aggressive and anxious/depressed behavior subscales of CBCL (Q), mother-report, mean score | OLS regression (including fixed-effects and residualized change models) | yes | yes | yes | yes | yes | yes |
|  | 2. change in weekly working hours between 3 and 5 years postpartum | 2. n/a |  |  |  |  |  |  |  |  |  |
| Fiori (2020) | 1. employed at 3 years postpartum  2. employed up to 15, 16–34, or 35+ hours at 3 years postpartum | 1. not employed at 3 years postpartum  2. not employed at 3 years postpartum | BP at 5 years | combination of hyperactivity/inatten-tion, emotional, conduct, and peer problems by use of SDQ (Q), reporting person not specified, cut-off for sum score used to define having BP | logistic regression | yes | yes | yes | yes | yes | no |
| **Study** | **Intervention** | **Control** | **Outcome** | **Outcome operationalization** | **Statistical method(s)** | **Adjusted for** | | | | | |
|  |  |  |  |  |  | sex^a^ | age^b^ | marital status^c^ | educa-tion^c^ | health^c^ | ethnicity^d^ |
| Gassman-Pines (2011) | 1. daytime working hours (08:00 a.m. to 06:00 p.m.)  2. night-time working hours (06:00 p.m. to 06:00 a.m.) | n/a | EBP, IBP, and positive behavior at preschool-age | EBP measured with 4 items of the IOWA Conners Rating Scale (Q), e.g., covering oppositional-defiant behavior; IBP measured with 5 items of the PBQ (Q), e.g., covering worrying; positive behavior measured with Positive Child Bids for Attention Scale (Q), covering positive bids for attention, mother-report, sum score | mixed models | yes | yes | yes | yes | no | yes |
| Goodman & Aber (2010) | employed (for at least 2 weeks) within past 6 months | not employed (for at least 6 weeks) within past 6 months | aggression (representational) at 3–5 years | child asked to finish five different doll stories by use of ASCT (I/O), sum score of aggression rating | hierarchical regression | yes | no | no | yes | yes | yes^f^ |
| Gupta & Simonsen (2007) | 1. degree of year mother was employed in 1996^e^  2. degree of year mother employed in 1997  3. degree of year mother employed in 1998 | n/a | BP at 7.5 years (in 2003) | combination of hyperactivity/inatten-tion, emotional, conduct, and peer problems by use of SDQ (Q), mother-report, sum score | OLS regression | yes | yes | yes | yes | yes | no |

| **Study** | **Intervention** | **Control** | **Outcome** | **Outcome operationalization** | **Statistical method(s)** | **Adjusted for** | | | | | |
| --- | --- | --- | --- | --- | --- | --- | --- | --- | --- | --- | --- |
|  |  |  |  |  |  | sex^a^ | age^b^ | marital status^c^ | educa-tion^c^ | health^c^ | ethnicity^d^ |
| Haas (2013) | usual weekly working hours | n/a | EBP at an approximate child age of 4 years | EBP subscale, e.g., covering aggressive and hyperactive behavior, of PKBS-2 (Q), parent- and caregiver-report, sum score | OLS regression | yes | no | no | yes | no | yes |
| Hadzic et al. (2013) | 16–34 weekly working hours at child age 4–5 years | 1. not working for pay  2. 1–15 weekly working hours  3. 35–40 weekly working hours  4. more than 40 weekly working hours | hyperactivity/inattention, conduct problems, and PB at child age 4–5 years | subscales of SDQ (Q), parent-report, sum score | two-wave panel mediation model | yes | no | yes^f^ | yes | no | no |
| Hall et al. (2008) | employed | not employed | EBP and IBP at child age 2–6 years | EBP subscale, i.e., covering attention problems and aggressive behavior, and IBP subscale, e.g., covering anxious/depressed and withdrawn behavior, of CBCL (Q), mother-report, sum score | multiple regression | yes | yes | yes^f^ | yes | yes | yes |

| **Study** | **Intervention** | **Control** | **Outcome** | **Outcome operationalization** | **Statistical method(s)** | **Adjusted for** | | | | | |
| --- | --- | --- | --- | --- | --- | --- | --- | --- | --- | --- | --- |
|  |  |  |  |  |  | sex^a^ | age^b^ | marital status^c^ | educa-tion^c^ | health^c^ | ethnicity^d^ |
| Hart & Kelley (2006) | weekly working hours (outside of home) | n/a | EPB and IBP at child age 1.5–4 years | EBP subscale, i.e., covering attention problems and aggressive behavior, and IBP subscale, e.g., covering anxious/depressed and withdrawn behavior, of CBCL (Q), mother- and father-report (averaged), sum score | multiple regression | no | no | yes^f^ | no | no | no |
| Herbst (2017) | 1. worked between childbirth and 9 months postpartum  2. number of months worked between childbirth and 9 months postpartum | 1. did not work between childbirth and 9 months postpartum  2. n/a | EBP, being unhappy, worrying, and PB at 60 months | subscales on EBP, i.e., aggressive and impulsive behavior, and PB, i.e., friendly and empathic behavior, of the PKBS-2 (Q), and single-item measures of being unhappy and worrying, teacher-report, sum scores where possible | IV regression | yes | yes | yes | yes | no | yes |
| Hill et al. (2005) | 1. not working within first year postpartum but working sometime in years 2–3 postpartum  2. working part-time in first year postpartum | 1. not working within first 3 years postpartum  2. not working within first year postpartum but working sometime in years 2–3 postpartum | EBP and IPB at child age 5–6 years | EBP subscale, e.g., covering antisocial behavior, hyperactivity, and peer problems, and IBP subscale, e.g., covering anxious/depressed and dependent behavior, of BPI (Q), parent-report, sum score | regression with and without propensity score matching | yes | no^k^ | yes | yes | no | yes |
| **Study** | **Intervention** | **Control** | **Outcome** | **Outcome operationalization** | **Statistical method(s)** | **Adjusted for** | | | | | |
|  |  |  |  |  |  | sex^a^ | age^b^ | marital status^c^ | educa-tion^c^ | health^c^ | ethnicity^d^ |
| Hill et al. (2005) | 3. working full-time in first year postpartum  4. working full-time in first year postpartum  5. working full-time in first year postpartum | 3. not working within first year postpartum but working sometime in years 2–3 postpartum  4. working part-time in first year postpartum  5. not working within first 3 years postpartum |  |  |  |  |  |  |  |  |  |
| Im & Vanderweele (2018) | employed in first year postpartum | not employed in first year postpartum | EBP and IBP at child ages 3 and 5 years | EBP subscale, i.e., covering aggressive and delinquent behavior, and IBP subscale, e.g., covering anxious/depressed and withdrawn behavior, of CBCL (Q), caregiver-report | regression with propensity score stratification and weighting | yes | yes | yes | yes | yes | yes |
| Jackson, Choi, & Bentler (2009) | average weekly working hours at child age 3–4 years | n/a | 1. BP at child age 3–4 years and 1.5–2 years later | Problem Behaviors Scale (Q), mother-report, mean | SEM | no | no | yes^f^ | yes | yes | yes^f^ |
| Jackson, Choi, & Franke (2009) | average weekly working hours at child age 3–4 years | n/a | BP 1.5–2 years after measurement of maternal working hours | Problem Behaviors Scale (Q), mother-report, mean | SEM | no | no | yes^f^ | yes | no | yes^f^ |
|  |  |  |  |  |  |  |  |  |  |  |  |
| **Study** | **Intervention** | **Control** | **Outcome** | **Outcome operationalization** | **Statistical method(s)** | **Adjusted for** | | | | | |
|  |  |  |  |  |  | sex^a^ | age^b^ | marital status^c^ | educa-tion^c^ | health^c^ | ethnicity^d^ |
| Jaursch & Lösel (2011) | 1. returning to work within first year postpartum  2. employed full-time within years 1–3 postpartum  3. employed full-time currently, i.e., at child age 2–6 | 1. returning to work in second or third year postpartum  2. employed part-time or not employed within years 1–3 postpartum  3. employed part-time or not employed currently | BP at 2–6 years | combination of hyperactivity/inatten-tion, aggression, delinquency, and emotional problems/ anxiousness, SBQ (Q), mother- and teacher-report (separately or averaged), sum score | ANCOVA | yes | no^j^ | no | no | no | no |
| Kiernan & Mensah (2009) | not employed at both 9 months and 3 years postpartum | 1. employed at 9 months postpartum only  2. employed at 3 years postpartum only  3. employed at both 9 months and 3 years postpartum | BP at 3 years | combination of hyperactivity/inatten-tion, emotional, conduct, and peer problems by use of SDQ (Q), mother-report, cut-off for sum score used to define having BP | logistic regression | yes | yes | yes | yes | yes | yes |

|  |  |  |  |  |  |  |  |  |  |  |  |
| --- | --- | --- | --- | --- | --- | --- | --- | --- | --- | --- | --- |
| **Study** | **Intervention** | **Control** | **Outcome** | **Outcome operationalization** | **Statistical method(s)** | **Adjusted for** | | | | | |
|  |  |  |  |  |  | sex^a^ | age^b^ | marital status^c^ | educa-tion^c^ | health^c^ | ethnicity^d^ |
| Law et al. (2014) | employed full-time at 3, 5, 7 years | 1. employed part-time at 3, 5, 7 years  2. not employed at 3, 5, 7 years | BP at 3, 5, 7 years | combination of hyperactivity/inatten-tion, emotional, conduct, and peer problems by use of SDQ (Q), main respondent-report, cut-off for sum score used to define having BP | regression | no | yes | yes | yes | no | yes |
| Le Zhang et al. (2020) | employed at 33 months | not employed at 33 months | BP at 42 months | combination of hyperactivity/inatten-tion, emotional, conduct, and peer problems by use of SDQ (Q), caregiver-report, sum score | regression | yes | no^j^ | yes | no | yes | no |
| Lekfuangfu et al. (2015) | 1. return to work at 0–6 months, 7–12 months, or 13–18 months postpartum  2. full-time or part-time employed at 18 months postpartum | 1. return to work after 18 months postpartum or not at all  2. return to work after 18 months postpartum or not at all | EBP, hyperactivity/ inattention, conduct problems, IBP, emotional problems, peer problems, and PB at 47 and 81 months | EBP subscale, i.e., covering hyperactivity/inattention, and conduct problems, IBP subscale, i.e., covering emotional and peer problems, and PB subscale of SDQ (Q), carer-report^q^, sum score | OLS regression | yes | yes | no^p^ | yes | yes | yes |

| **Study** | **Intervention** | **Control** | **Outcome** | **Outcome operationalization** | **Statistical method(s)** | **Adjusted for** | | | | | |
| --- | --- | --- | --- | --- | --- | --- | --- | --- | --- | --- | --- |
|  |  |  |  |  |  | sex^a^ | age^b^ | marital status^c^ | educa-tion^c^ | health^c^ | ethnicity^d^ |
| Lombardi & Coley (2014) | 1. return to employment before 9 months postpartum (part-time, full-time) | 1. return to employment between 9 and 24 months postpartum (part-time, full-time) or no return to employment within first 24 months postpartum | conduct problems, attention, and PB at kindergarten entry | items drawn from PKBS-2 (Q) and SSRS, additional items specifically created, teacher-report | OLS regression | yes | yes | yes | yes | yes | yes |
|  | 2. return to employment between 9 and 24 months postpartum (part-time, full-time) | 2. return to employment before 9 months postpartum or no return to employment within first 24 months postpartum (part-time, full-time) |  |  |  |  |  |  |  |  |  |
| Lombardi & Coley (2014) | 3. employed at outcome assessment | 3. not employed at outcome assessment |  |  |  |  |  |  |  |  |  |

| **Study** | **Intervention** | **Control** | **Outcome** | **Outcome operationalization** | **Statistical method(s)** | **Adjusted for** | | | | | |
| --- | --- | --- | --- | --- | --- | --- | --- | --- | --- | --- | --- |
|  |  |  |  |  |  | sex^a^ | age^b^ | marital status^c^ | educa-tion^c^ | health^c^ | ethnicity^d^ |
| McMunn et al. (2011) | number of sweeps mother was in paid work (maximum of 3 as employment was measured at child age 9 months, 3 years, and 5 years^g^) | n/a | BP at 5 years | combination of hyperactivity/inatten-tion, emotional, conduct, and peer problems by use of SDQ (Q), parent-report, cut-off for sum score used to define having BP | logistic regression^h^ | yes^i^ | yes | yes | yes | yes | yes^f^ |
| Meysamie et al. (2013) | employed | not employed (or having free job) | aggression (verbal physical, relational) at 3–6 years | scale developed by Shahim (2006^s^; Q), parent- and teacher-report, cut-off based on sum score to define aggressive and non-aggressive children | logistic regression | yes^t^ | no^u^ | yes^v^ | no | no | no |
| Miller-Lewis et al. (2006) | employed at child age 4 years | not employed at child age 4 years | EBP at 6 years | EBP subscale, i.e., covering attention problems and aggressive behavior, of CBCL/TRF (Q; depending on whether parent-, or teacher-report), cut-off for sum score used to define having EBP | logistic regression | yes | no^j^ | yes | yes | yes | no |

| **Study** | **Intervention** | **Control** | **Outcome** | **Outcome operationalization** | **Statistical method(s)** | **Adjusted for** | | | | | |
| --- | --- | --- | --- | --- | --- | --- | --- | --- | --- | --- | --- |
|  |  |  |  |  |  | sex^a^ | age^b^ | marital status^c^ | educa-tion^c^ | health^c^ | ethnicity^d^ |
| Nomaguchi (2006) | full-time year round or part-time/part year employed (measured at 2 and 4 years postpartum)^w^ | not employed in previous 12 months (measured at 2 and 4 years postpartum)^w^ | aggression (physical), hyperactivity, anxiety, unhappy mood, and PB at 2 and 4 years | n/a (Q), mother-report | OLS regression | yes | yes | yes | yes | no | no |
| Osborne & Knab (2007) | employed at 3 years postpartum | not employed at 3 years postpartum | aggressive, anxious/depressed, and withdrawn behavior at 3 years | subscales of CBCL (Q), mother-report, sum/mean score | n/a | no | no | no | no | no | no |
| Park et al. (2015) | working | not working | anxiety at 6–7 years | SAI as part of STAI-C (Q), child-report, sum score | Student's t-test (or Mann–Whitney U test for nonparametric data) | no | no | yes^f^ | no | no | no |
| Pekkurnaz (2014) | working hours in last week (measured at previous wave) | n/a | BP, attention, PB | factor analysis used to create indexes covering externalizing and internalizing behavior (BP index), attention, and PB, observer-/ parent-report, sum score | multiple regression (GMM) | yes | yes | yes | yes | no | yes |

| **Study** | **Intervention** | **Control** | **Outcome** | **Outcome operationalization** | **Statistical method(s)** | **Adjusted for** | | | | | |
| --- | --- | --- | --- | --- | --- | --- | --- | --- | --- | --- | --- |
|  |  |  |  |  |  | sex^a^ | age^b^ | marital status^c^ | educa-tion^c^ | health^c^ | ethnicity^d^ |
| Perry & Fantuzzo (2010) | persistence of unemployment within first 4 years postpartum | n/a | EBP, disruptive behavior, IBP, PB, and cooperative behavior at 4 years | EBP subscale, i.e., covering attention problems and aggressive behavior, and IBP subscale, e.g., covering anxious/depressed and withdrawn behavior, of CBCL (Q), disruptive behavior, PB and cooperative behavior measured by use of ASBI (Q), mother-report, sum score | hierarchical multiple regression | yes | yes | yes | yes | yes | yes |
| Perry-Jenkins et al. (2020) | working hours in first year postpartum | n/a | BP at 6 years | Behavioral Symptoms Index, e.g., covering aggression and depression, of BASC-PRS and BASC-TRS (Q), parent- and teacher-report combined | SEM | yes | no^k^ | yes | yes | no | no |

| **Study** | **Intervention** | **Control** | **Outcome** | **Outcome operationalization** | **Statistical method(s)** | **Adjusted for** | | | | | |
| --- | --- | --- | --- | --- | --- | --- | --- | --- | --- | --- | --- |
|  |  |  |  |  |  | sex^a^ | age^b^ | marital status^c^ | educa-tion^c^ | health^c^ | ethnicity^d^ |
| Philipsen Hetzner (2011) | working full-time or part-time at 9 months postpartum | not working at 9 months postpartum | BP at 2 years, EBP and PB at 4 years | BP covered attention, frustration, persistence, and social items from BRS (O), combination of interviewer- and parent-report, average score; EBP, e.g., covering aggressive and impulsive behavior, and PB, e.g., including friendly and comforting behavior, measured by PKBS-2 (Q) and SSRS (Q), mother-report | OLS regression with and without propensity score matching, SEM | yes | yes | yes | yes | yes/no^r^ | yes |
| Pilarz (2021; published online in 2020) | working full-time (35–40 hours) at 5 years postpartum | 1. working part-time (1–34 hours) at 5 years postpartum  2. working more than 40 hours at 5 years postpartum | aggression, IBP at 5 years | subscales, i.e., covering aggression and anxious/depressed, withdrawn behavior, of CBCL (Q), mother-report | OLS regression, lagged dependent variable model | yes | yes | yes | yes | yes | yes |
| Pilkauskas et al. (2017) | 1. number of months employed from birth to 5 years postpartum (and number of months employed within the same job) | 1. n/a | EBP, IBP at 5 years; changes in EBP between 3 and 5 years | EBP subscale, i.e., covering attention problems and aggressive behavior, and IBP subscale, i.e., covering anxious/depressed and withdrawn behavior, of CBCL (Q), mother-report, sum score | OLS regression | yes | yes | yes | yes | yes | yes |
| **Study** | **Intervention** | **Control** | **Outcome** | **Outcome operationalization** | **Statistical method(s)** | **Adjusted for** | | | | | |
|  |  |  |  |  |  | sex^a^ | age^b^ | marital status^c^ | educa-tion^c^ | health^c^ | ethnicity^d^ |
| Pilkauskas et al. (2017) | 2. employed 1–11, 12–23, 24–35, 36–47, 48–59, or 60+ months from birth to 5 years postpartum (and within the same job) | 2. no employment within first 5 years postpartum (and every other level of employment duration) |  |  |  |  |  |  |  |  |  |
|  | 3. number of months employed between 1 and 3 years postpartum | 3. n/a |  |  |  |  |  |  |  |  |  |
|  | 4. number of months employed between 3 and 5 years postpartum | 4. n/a |  |  |  |  |  |  |  |  |  |
|  | 5. changes in number of months employed between 1 and 3 as well as 3 and 5 years postpartum | 5. n/a |  |  |  |  |  |  |  |  |  |
|  | 6. number of months in longest job from birth to 5 years postpartum | 6. n/a |  |  |  |  |  |  |  |  |  |
| Rosenbaum & Morett (2009) | working part-time | working full-time | regulatory problems at 24 months, change in regulatory problems between 9 and 24 months | ITSC (Q), mother-report, mean score | OLS regression | yes | yes | yes^f^ | n/a | yes | yes |
| **Study** | **Intervention** | **Control** | **Outcome** | **Outcome operationalization** | **Statistical method(s)** | **Adjusted for** | | | | | |
|  |  |  |  |  |  | sex^a^ | age^b^ | marital status^c^ | educa-tion^c^ | health^c^ | ethnicity^d^ |
| Strazdins et al. (2010) | average weekly working hours | n/a | BP at 4–5 years | combination of hyperactivity/inatten-tion, emotional, conduct, and peer problems by use of SDQ (Q), primary caregiver-report, sum score | multiple linear regression | yes | no^k^ | yes | yes | yes | no |
| Strazdins et al. (2013) | average weekly working hours | n/a | BP at 4–5 years | combination of hyperactivity/inatten-tion, emotional, conduct, and peer problems by use of SDQ (Q), primary caregiver-report, sum score | hierarchical linear regression | yes | no^k^ | yes | yes | yes | no |
| Sugawara (2005) | 1. returned to work within first 3 years postpartum  2. employed full-time | did not return to work within first 3 years postpartum  2. employed part-time | EBP at 5 years | EEPBCL (Q), mother-report, sum score | t-test | no | no | no | no | no | no |
| Sundaur & Rustina (2019) | employed | not employed | BP at 3–6 years | KMME (Q), mother-report, cut-off used to define emotional deviation | χ^2^-test | no | no | no | no | no | no |

| **Study** | **Intervention** | **Control** | **Outcome** | **Outcome operationalization** | **Statistical method(s)** | **Adjusted for** | | | | | |
| --- | --- | --- | --- | --- | --- | --- | --- | --- | --- | --- | --- |
|  |  |  |  |  |  | sex^a^ | age^b^ | marital status^c^ | educa-tion^c^ | health^c^ | ethnicity^d^ |
| Turney (2012) | employed at 5 years postpartum | not employed at 5 years postpartum | EBP, IBP at 5 years | EBP subscale, i.e., covering attention problems and aggressive behavior, and IBP subscale, i.e., covering anxious/depressed and withdrawn behavior, of CBCL (Q), mother-report, sum score | OLS regression | yes | yes | yes | yes | yes | yes |
| Whitaker et al. (2006) | employed for 12 months within first year postpartum | employed for <1, 1–6, or 7–11 months within first year postpartum | aggressive, anxious/depressed behavior, and inattention/hyper-activity at 3 years | subscales (aggressive and anxious/depressed behavior) and selected items (by professionals; inattention/hyperactivity) of CBCL (Q), mother-report, cut-off used to define problem behavior | logistic regression | no | yes | yes | yes | yes | yes |
| Yoldaş et al. (2020) | working | not working | IBP, anxious/depressed behavior at 2–5 years | subscales (anxious/depressed behavior, IBP subscale) of CBCL (Q), parent-report | Student's t-test (or Mann–Whitney U test for nonparametric data) | no | no | no | no | no | no |

EBP = externalizing behavior problems; BPI = Behavioural Problems Index; Q = questionnaire; OLS = ordinary least squares; BP = behavior problems; CBCL 1½–5 = Child Behavior Checklist 1½–5;

SEM = structural equation modeling; n/a = not available/applicable; SDQ = Strengths and Difficulties Questionnaire; IBP = internalizing behavior problems; IOWA = Inattention/Overactivity with Aggression; PBQ = Preschool Behavior Questionnaire; ASCT = Attachment Story-Completion Task; I = interview; O = observation; PKBS-2 = Preschool and Kindergarten Behavior Scales – Second Edition; PB = prosocial behavior; IV = Instrumental Variables; SBQ = Social Behavior Questionnaire; ANCOVA = Analysis of covariance; SSRS = Social Skills Rating Scale; TRF = Teacher Report Form; SAI = State Anxiety Inventory; STAI-C = State-Trait Anxiety Inventory-Child Version; GMM = Generalized Method of Moments; ASBI = Adaptive Social Behavior Inventory; BASC-PRS = Behavioral Assessment System for Children – Parent Rating Scale; BASC-TRS = Behavioral Assessment System for Children – Teacher Rating Scale; BRS = Behavior Rating Scale; ITSC = Infant/Toddler Symptoms Checklist; EEPBCL = Emergence of Externalizing Problem Behavior Check List; KMME = Kuesioner Masalah Mental Emotional.

^a^Of children. ^b^Of both mothers and children (labelled “yes” for children even in case age was not controlled for but was unlikely to vary markedly). ^c^Of mothers. ^d^Either of mothers or children. ^e^Children were born in 1995. ^f^Due to inclusion criteria. ^g^In further, but unadjusted analyses full-time employment was compared to part-time employment or no employment at child age 9 months. ^h^Further, but unadjusted analyses relied on cross-tabulations. ^I^Due to subgroup analyses. ^j^Adjusted for child age only. ^k^Adjusted for maternal age only. ^l^In further, but unadjusted analyses employment was compared to no employment at 15, 24, or 36 months, and between months 42 and 54. ^m^Further, but unadjusted analyses relied on correlations. ^n^Further, but unadjusted analyses (only controlling for ethnicity due to inclusion criteria) relied on correlations. ^o^Some information on outcome assessment at 3 years missing but inferred from information on study provided by NICHD itself. ^p^Only in single subgroup analyses. ^q^Values on the 4 subscales hyperactivity/inattention, emotional, peer, and conduct problems were also derived by teacher-report at child age 81 months. ^r^Yes for age 2 years outcome, no for age 4 years outcomes. ^s^ Shahim S. Overt and relational aggression among elementary school children. *Psychol Res* 2006;**9**:27-44. ^t^For physical aggression only. ^u^Adjusting for child age when focus on physical aggression, adjusting for maternal age when focus on relational aggression. ^v^For relational aggression only. ^w^In further, but unadjusted analyses children of mothers employed full-time year round were compared to children whose mothers were employed part-time/part year.

| **Supplementary Table S3.** Risk of bias assessment. | | | | | | | | | |
| --- | --- | --- | --- | --- | --- | --- | --- | --- | --- |
|  | | | | | | | | | |
| **Study** | **Major domains** | | | | | **Minor domains** | | | **Overall risk of bias** |
|  | Recruitment procedure and follow-up^a^ | Exposure^b^ | Outcome | Confounding, effect modification | Analysis method(s) | Chronology | Funding | Conflict of interest |  |
| Berger et al. (2005) | UR | LR | LR | HR | LR/HR | LR | LR | UR | **HR** |
| Berger et al. (2008) | LR | LR | LR | LR | LR | LR | LR | UR | **LR** |
| Brooks-Gunn et al. (2010) | HR | LR | LR | LR | UR | LR | LR | UR | **HR** |
| Cabrera et al. (2014) | UR | LR | LR | HR | LR | LR | LR | UR | **HR** |
| Conway et al. (2017) | UR | LR | LR | LR | LR | LR | LR | LR | **MR** |
| Deding et al. (2007) | LR | HR | LR | HR | HR | LR | UR | UR | **HR** |
| Dunifon et al. (2013) | LR | LR | LR | LR | LR | LR | LR | UR | **LR** |
| Fiori (2020) | HR | UR | UR | HR | LR | LR | LR | UR | **HR** |
| Gassman-Pines (2011) | LR | LR | HR | HR | LR | LR | UR | UR | **HR** |
| Goodman & Aber (2010) | UR | HR | LR | HR | HR | LR | LR | UR | **HR** |
| Gupta & Simonsen (2007) | LR | UR | LR | HR | HR | LR | HR | HR | **HR** |
| **Study** | **Major domains** | | | | | **Minor domains** | | | **Overall risk of bias** |
|  | Recruitment procedure and follow-up^a^ | Exposure^b^ | Outcome | Confounding, effect modification | Analysis method(s) | Chronology | Funding | Conflict of interest |  |
| Haas (2013) | LR | LR | LR | HR | LR | LR | UR | UR | **MR** |
| Hadzic et al. (2013) | LR | LR | LR | HR | HR | LR | UR | UR | **HR** |
| Hall et al. (2008) | LR | LR | LR | LR | LR | LR | LR | UR | **LR** |
| Hart & Kelley (2006) | HR | LR | LR | HR | UR | LR | UR | UR | **HR** |
| Herbst (2017) | HR | UR | LR | HR | UR | LR | UR | UR | **HR** |
| Hill et al. (2005) | UR | LR | LR | HR | LR | LR | LR | UR | **HR** |
| Im & Vanderweele (2018) | UR | LR | LR | LR | LR | LR | LR | LR | **MR** |
| Jackson, Choi, & Bentler (2009) | LR | LR | UR | HR | HR | LR | LR | UR | **HR** |
| Jackson, Choi, & Franke (2009) | LR | LR | UR | HR | HR | UR | LR | UR | **HR** |
| Jaursch & Lösel (2011) | LR | LR | LR | HR | HR | LR | LR | UR | **HR** |
| Kiernan & Mensah (2009) | LR | LR | LR | LR | LR | LR | LR | UR | **LR** |
| Law et al. (2014) | LR | LR | LR | HR | HR | LR | LR | UR | **HR** |
| Le Zhang et al. (2020) | HR | LR | LR | HR | HR | LR | LR | LR | **HR** |
| Lekfuangfu et al. (2015) | HR | LR | LR | LR/HR | LR | LR | LR | UR | **MR/HR** |
| **Study** | **Major domains** | | | | | **Minor domains** | | | **Overall risk of bias** |
|  | Recruitment procedure and follow-up^a^ | Exposure^b^ | Outcome | Confounding, effect modification | Analysis method(s) | Chronology | Funding | Conflict of interest |  |
| Lombardi & Coley (2014) | HR | HR | UR | LR | LR | LR | LR | UR | **HR** |
| McMunn et al. (2011) | LR | HR | LR | LR | LR | LR | LR | LR | **MR** |
| Meysamie et al. (2013) | UR | HR | LR | HR | HR | LR | LR | LR | **HR** |
| Miller-Lewis et al. (2006) | LR | UR | LR | HR | UR | LR | UR | UR | **HR** |
| Nomaguchi (2006) | HR | LR | UR | HR | LR | LR | UR | UR | **HR** |
| Osborne & Knab (2007) | LR | LR | LR | HR | HR | LR | UR | UR | **HR** |
| Park et al. (2015) | UR | UR | LR | HR | HR | LR | UR | LR | **HR** |
| Pekkurnaz (2014) | HR | LR | UR | HR | HR | LR | UR | UR | **HR** |
| Perry & Fantuzzo (2010) | HR | LR | LR | LR | UR | LR | UR | UR | **HR** |
| Perry-Jenkins et al. (2020) | HR | LR | LR | HR | LR | LR | LR | UR | **HR** |
| Philipsen Hetzner (2011) | LR | LR | LR | LR/HR | LR | LR | UR | UR | **MR** |
| Pilarz (2021; published online in 2020) | LR | LR | LR | LR | LR | LR | UR | UR | **MR** |
| **Study** | **Major domains** | | | | | **Minor domains** | | | **Overall risk of bias** |
|  | Recruitment procedure and follow-up^a^ | Exposure^b^ | Outcome | Confounding, effect modification | Analysis method(s) | Chronology | Funding | Conflict of interest |  |
| Pilkauskas et al. (2017) | LR | LR | LR | LR | LR | LR | UR | UR | **MR** |
| Rosenbaum & Morett (2009) | LR | LR | LR | UR | LR | LR | UR | UR | **MR** |
| Strazdins et al. (2010) | LR | LR | LR | HR | LR | LR | LR | UR | **MR** |
| Strazdins et al. (2013) | LR | LR | LR | HR | LR | LR | LR | UR | **MR** |
| Sugawara (2005) | HR | LR | UR | HR | HR | UR | UR | UR | **HR** |
| Sundaur & Rustina (2019) | HR | LR | LR | HR | HR | LR | LR | LR | **HR** |
| Turney (2012) | UR | LR | LR | LR | HR | LR | UR | UR | **HR** |
| Whitaker et al. (2006) | HR | LR | LR/HR | UR | UR | LR | LR | UR | **HR** |
| Yoldaş et al. (2020) | UR | HR | LR | HR | HR | LR | LR | LR | **HR** |

UR = unclear risk of bias; LR = low risk of bias; HR = high risk of bias; MR = moderate risk of bias.

^a^If applicable. ^b^Including both intervention and comparison.

| **Supplementary Table S4.** Extracted results of studies assessing the association of maternal employment and child overall behavior problems. | | | |
| --- | --- | --- | --- |
|  | | | |
| **Study** | **General findings** | **Moderator/subgroup analyses** | **Mediator analyses** |
| Berger et al. (2008) | **no association** in OLS regression models without propensity score matching | association differs by **race/ethnicity**: in both OLS regression models, Hispanic children of working mothers exhibit more BP than children of mothers not working, in OLS regression models with propensity score matching only, Black children of working mothers exhibit less BP than children of mothers not working | additional inclusion of various so-called mediating variables, i.e., child care, parenting, and maternal depressive symptoms, did not alter results substantively; no further analyses of indirect or total effects |
| Deding et al. (2007) | n/a | **no associations** in both girls and boys | n/a |
| Fiori (2020) | **association found**: employment linked to lower likelihood of BP, especially when working 16­–34 or 35+ hours | n/a | additional inclusion of mediating variables, i.e., child care, household income, and maternal mental health, did not alter results substantively, with mediation found for household income and maternal mental health |
| Gupta & Simonsen (2007) | **no associations** regardless of year under investigation | n/a | n/a |
| Jackson, Choi, & Bentler (2009) | **negative cross-sectional association** in SEM analyses (direct and total effect): more working hours were linked to less BP;  **negative longitudinal association** in SEM analyses (total effect, no direct effect): more working hours were linked to less BP | n/a | **indirect effect** both cross-sectionally and longitudinally under consideration of various variables, e.g., earnings, maternal depressive symptoms, and parenting efficacy: more working hours were linked to less BP |
| Jackson, Choi, & Franke (2009) | **no association** in SEM analyses (no direct or total effect) | n/a | **no indirect effect** under consideration of various variables, e.g., income, parenting stress, and quality of mother-father relationship |
| Jaursch & Lösel (2011) | **no associations** regardless of intervention/control under investigation | **no interaction** of early maternal employment, i.e., within years 1–3 postpartum, and current maternal employment | n/a |
| Kiernan & Mensah (2009) | **association found**: children of mothers employed at both 9 months and 3 years postpartum with lower odds of experiencing BP compared to children whose mothers were not employed at both 9 months and 3 years postpartum;  children of mothers employed at either 9 months or 3 years postpartum did not differ from children whose mothers were not employed at both 9 months and 3 years postpartum | n/a | n/a |

| **Study** | **General findings** | **Moderator/subgroup analyses** | **Mediator analyses** |
| --- | --- | --- | --- |
| Law et al. (2014) | **association found**: employment linked to less BP | n/a | n/a |
| Le Zhang et al. (2020) | **association found**: employment linked to less BP | n/a | n/a |
| McMunn et al. (2011) | n/a | association differs by **sex**: girls whose mothers were not in paid work at any sweep with higher odds of having BP compared to girls whose mothers were in paid work at every sweep, no associations in boys^a^ | n/a |
| Pekkurnaz (2014) | **no association** | n/a | n/a |
| Perry-Jenkins et al. (2020) | **no association** | n/a | inclusion of parenting did not alter results |
| Philipsen Hetzner (2011) | **no associations** in both OLS regression models with and without propensity score matching | n/a | **no direct effect** (with inclusion of various mediating variables, i.e., quality of home environment, child care, breastfeeding, maternal knowledge of child development, depression, income, and time spent with child) |
| Strazdins et al. (2010) | **no association** | **no association** in both lone and coupled mothers, low-income and mid-to-high income mothers | parental distress |
| Strazdins et al. (2013) | **no association** | **no association** in dual-earner families | n/a |
| Sundaur & Rustina (2019) | **no association** | n/a | n/a |
| **Regulatory problems** |  |  |  |
| Rosenbaum & Morett (2009) | **no associations** | n/a | n/a |

OLS = ordinary least squares; BP = behavior problems; n/a = not available/applicable; SEM = structural equation modeling.

^a^ In further, but unadjusted analyses associations emerged, with both girls and boys of mothers being in paid work at child age 9 months with lower likelihood of exhibiting BP at child age 5 years.

| **Supplementary Table S5.** Extracted results of studies assessing the association of maternal employment and child externalizing behavior problems. | | | |
| --- | --- | --- | --- |
|  | | | |
| **Study** | **General findings** | **Moderator/subgroup analyses** | **Mediator analyses** |
| Berger et al. (2005) | returning to work within 12 weeks vs. not returning to work within 12 weeks: **no association** in OLS regression models without propensity score matching;  **association found** in OLS regression models with propensity score matching: returning to work was linked to more EBP  returning to work full-time within 12 weeks vs. not returning to work within 12 weeks: some evidence of an **association found** in both OLS regression models: returning to work full-time was linked to more EBP  returning to work full-time within 12 weeks vs. returning to work part-time within 12 weeks: **no association** | n/a | n/a |
| Brooks-Gunn et al. (2010) | n/a | associations differ by **ethnicity**:  full-time vs. no work by 12 months: at both child ages irrespective of use of mother- or caregiver-report **no association** in both White and Black children;  part-time vs. no work by 12 months: at both child ages irrespective of use of mother- or caregiver-report **no association** in both White and Black children;  full-time vs. part-time work by 12 months: some evidence of **association found** in White children with full-time work linked to more EBP (mother- and caregiver-report at child age 3 years, caregiver-report at child age 4.5 years), limited evidence of **association found** in Black children with part-time work linked to more EBP (mother-report at child age 4.5 years)^a^  child sex and temperament do not act as moderators (analysed for White children only) | **SEM models** (for White children only) **do not point to any direct, indirect, or total effects** of full-time or part-time work by 12 months (vs. no work) with inclusion of potentially mediating variables, i.e., maternal earnings, depressive symptoms, and sensitivity, quality of home environment, and type and quality of child care (irrespective of child age and reporting person);  direct, indirect, and total effect of full-time work by 12 months on EBP at child age 3 years larger than effects of part-time work (caregiver-report); indirect, and total effect of full-time work by 12 months on EBP at child age 4.5 years larger than effects of part-time work (mother- and caregiver-report with the latter including direct effect as well) |

| **Study** | **General findings** | **Moderator/subgroup analyses** | **Mediator analyses** |
| --- | --- | --- | --- |
| Brooks-Gunn et al. (2010) |  | associations differ by **maternal occupation** (professional vs. non-professional; analysed for White children only): associations of full-time work by 12 months (vs. part-time work) and more EBP at 3 years (caregiver-report) and 4.5 years (mother-report) larger for children of non-professional mothers  **timing of return to work** analyses (White children): full-time work by 3, 6, or 9 months (vs. no work) linked to more EBP at child age 4.5 years (caregiver-report), full-time work by 3 months (vs. part-time work) linked to more EBP at child age 3 years (mother-report) and 4.5 years (caregiver-report), full-time work by 6 months (vs. part-time work) linked to more EBP at child age 4.5 years (caregiver-report), full-time work by 9 months (vs. part-time work) linked to more EBP at child ages 3 years and 4.5 years (caregiver-report in both cases); at 12 months postpartum full-time and part-time work differed (with full-time work linked to more EBP) only when mothers had returned to work within 6 months postpartum (irrespective of reporting person at child age 3 years, caregiver-report at 4.5 years) |  |
| Cabrera et al. (2014) | **no associations** | association differs by **self-regulation** of the child: in less self-regulated children more maternal working hours were linked to less EBP at child age 4 years but also to a sharper increase in EBP between child ages 4 and 6 years, no associations in more self-regulated children (both coefficients differed across groups of self-regulation) | n/a |
| Conway et al. (2017) | **no associations** found at 4.5 years (mother-report, no results provided for caregiver-report), at child age 3 years **association found**: working part-time was both negatively and positively associated with EBP (depending on other variables included in the model; no results provided for working full-time)^b^ | n/a | **maternal depressive symptoms**: both full-time and part-time work were linked to more depressive symptoms at child age 3 years that in turn were associated with more EBP at child age 4.5 years (significance of indirect and total effect not reported) |

| **Study** | **General findings** | **Moderator/subgroup analyses** | **Mediator analyses** |
| --- | --- | --- | --- |
| Gassman-Pines (2011) | **no associations** | association does not differ by marital status (single vs. living with partner; tested for night-time working hours only) and type of day mother worked (weekdays vs. weekends; tested for night-time working hours only) | n/a |
| Haas (2013) | **association found** for caregiver-reported EBP only: more maternal working hours were linked to more EBP | **no interaction of maternal work schedule** and work hours (both mother- and caregiver-report) | n/a |
| Hall et al. (2008) | **no association** | n/a | n/a |
| Hart & Kelley (2006) | **no association** | n/a | n/a |
| Herbst (2017) | **no associations** | n/a | n/a |
| Hill et al. (2005) | **no associations** when comparing not working within first year postpartum but working sometime in years 2–3 postpartum and not working within first 3 years postpartum, working part-time in first year postpartum and not working within first year postpartum but working sometime in years 2–3 postpartum, working full-time in first year postpartum and not working within first year postpartum but working sometime in years 2–3 postpartum;  **association found** when comparing working full-time and working part-time in first year postpartum: working full-time was linked to more EBP (effect of treatment on treated, children of full-time working mothers exhibited more EBP than they would have had had their mothers worked part-time instead, in regression model without propensity score matching only);  **association found** when comparing working full-time in first year postpartum and not working within first 3 years postpartum: working full-time was linked to more EBP (effect of treatment on control, children of mothers not working would have exhibited more EBP had their mothers worked full-time instead, in regression models with and without propensity score matching only) | n/a | n/a |

| **Study** | **General findings** | **Moderator/subgroup analyses** | **Mediator analyses** |
| --- | --- | --- | --- |
| Im & Vanderweele (2018) | **no association** at child age 3 years;  **association found** at child age 5 years linking employment to more EBP | n/a | n/a |
| Lekfuangfu et al. (2015) | **association found** at child age 47 months linking full-time employment to more EBP^c^ (no further associations at that child age);  **associations found** at child age 81 months linking return to work between months 7–12^c^ and full-time employment to more EBP (no further associations at that child age) | associations differ by **sex**: in boys, only one association emerged linking full-time employment to more EBP at child age 81 months; this finding is replicated in girls but augmented by further associations linking returning to work between months 0–6 to less EBP at 47 months and between months 7–12 to more EBP at 81 months;  association differs by **child care** (centre-based vs. relative vs. commercial child care): both part-time and full-time employment linked to less EBP at 81 months when in centre-based child care (replicated for full-time employment at 47 months; tested for full-time or part-time employment at 18 months postpartum vs. return to work after 18 months postpartum or not at all only);  marital status (living with partner vs. not living with partner at 8 months postpartum), maternal education (completed at least high school vs. did not complete high school), and mental health did not moderate association (all tested for full-time or part-time employment at 18 months postpartum vs. return to work after 18 months postpartum or not at all only) | n/a |
| Miller-Lewis et al. (2006) | **no associations** for both parent- and teacher-reported EBP | n/a | n/a |
| Perry & Fantuzzo (2010) | **no association** | n/a | n/a |
| Philipsen Hetzner (2011) | **no associations** in both OLS regression models with and without propensity score matching | n/a | **no direct effect** (with inclusion of various mediating variables, i.e., quality of home environment, attachment, child care, child well visits, maternal sensitivity, income, and time spent with child) |

| **Study** | **General findings** | **Moderator/subgroup analyses** | **Mediator analyses** |
| --- | --- | --- | --- |
| Pilkauskas et al. (2017) | 1. number of months employed from birth to 5 years postpartum (+ within same job): **association** **found** with more months (in same job) linked to less EBP (but not in every robustness check)  2. employed 1–11, 12–23, 24–35, 36–47, 48–59, or 60+ months (+ within same job): employed for 48–59 months linked to less EBP (compared to no employment), coefficient of 48–59 months and 60+ months differed from those of other months categorizations  3. number of months employed between 1 and 3 years postpartum: **association found** linking more months to less EBP  4. number of months employed between 3 and 5 years postpartum: **no association**  5. changes in number of months employed between 1 and 3 as well as 3 and 5 years postpartum (on changes in behavior): **no association**  6. number of months in longest job from birth to 5 years postpartum: **association found** linking more months to less EBP |  |  |
| Sugawara (2005) | **association found** linking having returned to work to less EBP **(unadjusted analysis only)**  **no association** when comparing full-time and part-time employed mothers **(unadjusted analysis only)** | n/a | n/a |
| Turney (2012) | **no association** | n/a | n/a |
| **Aggression** |  |  |  |
| Dunifon et al. (2013) | **no associations** | n/a | inclusion of harsh parenting did not alter results |
| Goodman & Aber (2010) | **association found** linking employment to more aggression | association differed by **social support**: children of employed mothers showed less aggression in case of higher social support (no results for children of unemployed mothers);  sex did not moderate association (no interaction effect), however, employment was linked to more aggression in girls (no association for boys) | n/a |

| **Study** | **General findings** | **Moderator/subgroup analyses** | **Mediator analyses** |
| --- | --- | --- | --- |
| Meysamie et al. (2013) | **associations found** linking being employed to higher odds of physical aggression (parent-report only) and relational aggression (teacher-report only)  **no association** for verbal aggression | n/a | n/a |
| Nomaguchi (2006) | 2 years: **no association**  4 years: full-time linked to lower aggression cross-sectionally, full-time and part-time linked to higher aggression longitudinally^d^ | n/a | inclusion of potentially mediating variables, i.e., mother-child interaction, child care, hours in school settings, did not alter results |
| Osborne & Knab (2007) | **no associations** (unadjusted analyses only) | n/a | n/a |
| Pilarz (2021; published online in 2020) | **association found** linking part-time employment to less aggression  **no association** when comparing full-time employment and working more than 40 hours | n/a | n/a |
| Whitaker et al. (2006) | **no associations** | n/a | n/a |
| **Disruptive behavior** | | | |
| Perry & Fantuzzo (2010) | **no association** | n/a | n/a |
| **Hyperactivity/inattention** | | | |
| Hadzic et al. (2013) | **direct effect** linking 35–40 hours to more hyperactivity/inattention | n/a | **indirect effects** linking 1–15 and more than 40 working hours to less hyperactivity inattention via maternal parenting practices |
| Lekfuangfu et al. (2015) | **associations found** linking full-time employment to more hyperactivity/inattention at both child ages (carer-report; no further associations) | n/a | n/a |
| Lombardi & Coley (2014) | **no associations** | ethnicity did not moderate association, no interaction effects of return to work (before 9 months, between 9 and 24 months) and hours in nonparental care, maternal wages, depressive symptoms, and non-maternal household income | inclusion of hours in nonparental care, maternal wages, and depressive symptoms did not alter results |
| Nomaguchi (2006) | 2 years: **no association**  4 years: **no** cross-sectional or longitudinal **associations**^e^ | n/a | 2 years: inclusion of potentially mediating variables, i.e., mother-child interaction, child care, hours in school settings, did not alter results  4 years: cross-sectionally, part-time linked to lower hyperactivity; longitudinally, results did not change |
| Pekkurnaz (2014) | **no association** | n/a | n/a |
| Whitaker et al. (2006) | **no associations** | n/a | n/a |
| **Study** | **General findings** | **Moderator/subgroup analyses** | **Mediator analyses** |
| **Conduct problems** | | | |
| Hadzic et al. (2013) | **no direct effects** | n/a | **indirect effects** linking 1–15 and more than 40 working hours to less conduct problems via maternal parenting practices |
| Lekfuangfu et al. (2015) | **associations found** linking returning to work at 7–12 months postpartum to more conduct problems at child age 81 months (carer-report) and full-time employment to more conduct problems at both child ages (carer-report; no further associations) | n/a | n/a |
| Lombardi & Coley (2014) | **association found** linking return to employment between 9 and 24 months to more conduct problems (compared to return to employment before 9 months), returning to work between 9 and 24 months part-time was linked to more conduct problems (compared to no employment, returning full-time or having returned before 9 months, either part-time or full-time), no further associations | ethnicity did not moderate association, no interaction effects of return to work (before 9 months, between 9 and 24 months) and hours in nonparental care, maternal wages, and depressive symptoms;  association differed by **non-maternal household income**: returning to employment between 9 and 24 months was linked to more conduct problems as household income increased | after inclusion of hours in nonparental care, maternal wages, and depressive symptoms return to work analyses did no longer yield results |

OLS = ordinary least squares; EBP = externalizing behavior problems; n/a = not available/applicable.

^a^In further, but unadjusted analyses associations emerged, with employment at 24 months linked to lower EBP at child age 3 years (mother-report) and more EBP at child age 4.5 years (caregiver-report), being employed between months 42 and 54 linked to more EBP at child age 4.5 years (caregiver-report); employment at 15/36 months not associated with EBP (irrespective of child age and reporting person). ^b^In further, but unadjusted analyses (only controlling for ethnicity due to inclusion criteria) associations were found for caregiver-reported EBP, with full-time employment linked to more EBP and part-time employment linked to less EBP. ^c^Association turns out to be insignificant in robustness checks including paternal time inputs (however, could also be attributed to smaller sample size). ^d^In further, but unadjusted analyses part-time/part year employment was linked to more aggression at child age 4 years (both cross-sectionally and longitudinally) compared to full-time year round employment (no difference at child age 2 years). ^e^In further, but unadjusted analyses no differences between part-time/part year employment full-time year round employment emerged.

| **Supplementary Table S6.** Extracted results of studies assessing the association of maternal employment and child internalizing behavior problems. | | | |
| --- | --- | --- | --- |
|  | | | |
| **Study** | **General findings** | **Moderator/subgroup analyses** | **Mediator analyses** |
| Gassman-Pines (2011) | **no associations** | association does not differ by marital status (single vs. living with partner; tested for night-time working hours only) and type of day mother worked (weekdays vs. weekends; tested for night-time working hours only) | n/a |
| Hall et al. (2008) | **no association** | n/a | n/a |
| Hart & Kelley (2006) | **no association** | n/a | n/a |
| Hill et al. (2005) | **no associations** | n/a | n/a |
| Im & Vanderweele (2018) | **association found** at child age 3 years linking employment to more IBP;  **no association** at child age 5 years | association differs by **paternal involvement** at child age 3 years: employment linked to more IBP under low paternal involvement, employment linked to less IBP under high paternal involvement; employment linked to more IBP under absent paternal involvement, no difference under present paternal involvement | n/a |
| Lekfuangfu et al. (2015) | **no associations** at child age 47 months, only at 81 months **association found** linking returning to work at 13–18 months postpartum to less IBP (no further associations at that child age) | association differs by **child care** (centre-based vs. relative vs. commercial child care): full-time employment linked to less IBP at 81 months when in centre-based or commercial child care (tested for full-time or part-time employment at 18 months postpartum vs. return to work after 18 months postpartum or not at all only);  associations do not differ by sex as in both girls and boys no associations remain (at both child ages);  marital status (living with partner vs. not living with partner at 8 months postpartum), maternal education (completed at least high school vs. did not complete high school), and mental health did not moderate association (all tested for full-time or part-time employment at 18 months postpartum vs. return to work after 18 months postpartum or not at all only) | n/a |

| **Study** | **General findings** | **Moderator/subgroup analyses** | **Mediator analyses** |
| --- | --- | --- | --- |
| Perry & Fantuzzo (2010) | **association found** linking longer lasting unemployment to more IPB | n/a | n/a |
| Pilarz (2021; published online in 2020) | **no associations** | n/a | n/a |
| Pilkauskas et al. (2017) | 1. number of months employed from birth to 5 years postpartum (+ within same job): **no association**  2. employed 1–11, 12–23, 24–35, 36–47, 48–59, or 60+ months (+ within same job): **no association**, however, coefficient for 48–59 months differs from those for less than 36 months (less IBP)  3. number of months employed between 1 and 3 years postpartum: **no association**  4. number of months employed between 3 and 5 years postpartum: **no association**  5. changes in number of months employed between 1 and 3 as well as 3 and 5 years postpartum (on changes in behavior): **no association**  6. number of months in longest job from birth to 5 years postpartum: **no association** |  |  |
| Turney (2012) | **no associations** | n/a | n/a |
| Yoldaş et al. (2020) | **association found** linking employment to less IBP | n/a | n/a |
| **Anxious/depressed behavior** | | | |
| Dunifon et al. (2013) | **no associations** | n/a | inclusion of harsh parenting did not alter results |
| Nomaguchi (2006) | 2 years: **no association**  4 years: **no** cross-sectional or longitudinal **associations**^a^ | n/a | 2 years: inclusion of potentially mediating variables, i.e., mother-child interaction, child care, hours in school settings, did not alter results  4 years: cross-sectionally, full-time and part-time linked to lower anxiety; longitudinally, results did not change |
| Osborne & Knab (2007) | **no associations** (unadjusted analyses only) | n/a | n/a |
| Park et al. (2015) | **no association** | n/a | n/a |
| Whitaker et al. (2006) | **association found** linking maternal employment of less than 1 month duration to higher odds of having problematic behavior, no further associations | n/a | n/a |
| Yoldaş et al. (2020) | **association found** linking employment to less anxious/  depressed behavior | n/a | n/a |

| **Study** | **General findings** | **Moderator/subgroup analyses** | **Mediator analyses** |
| --- | --- | --- | --- |
| **Emotional problems** | | | |
| Lekfuangfu et al. (2015) | only at 81 months **association found** linking returning to work at 7–12 months postpartum to less emotional problems (teacher-report) | n/a | n/a |
| **Being unhappy** | | | |
| Herbst (2017) | **no associations** | n/a | n/a |
| Nomaguchi (2006) | 2 years: **no association**  4 years: **no** cross-sectional or longitudinal **associations**^b^ | n/a | inclusion of potentially mediating variables, i.e., mother-child interaction, child care, hours in school settings, did not alter results |
| **Worrying** | | | |
| Herbst (2017) | **no associations** | n/a | n/a |
| **Peer problems/withdrawn behavior** | | | |
| Lekfuangfu et al. (2015) | only at 81 months **association found** linking returning to work at 7–12 months postpartum to less peer problems (carer- and teacher-report; no further associations) | n/a | n/a |
| Osborne & Knab (2007) | **no associations** (unadjusted analyses only) | n/a | n/a |

n/a = not available/applicable; IBP = internalizing behavior problems.

^a^In further, but unadjusted analyses part-time/part year employment was linked to less anxiety at child age 2 (no further association. ^b^In further, but unadjusted analyses no differences between part-time/part year employment full-time year round employment emerged.

| **Supplementary Table S7.** Extracted results of studies assessing the association of maternal employment and child positive, prosocial, or cooperative behavior. | | | |
| --- | --- | --- | --- |
|  | | | |
| **Study** | **General findings** | **Moderator/subgroup analyses** | **Mediator analyses** |
| Gassman-Pines (2011) | **association found** for night-time working hours only: more working hours were linked to less positive behavior that day (effects of night-time and daytime working hours did not differ indicating that association is not specific to night-time working hours and number of working hours per se seems to matter instead) | association differs by **type of day mother worked** (weekdays vs. weekends, tested for night-time working hours only): larger negative association found for night-time working hours on weekends;  association does not differ by marital status (single vs. living with partner; tested for night-time working hours only) | n/a |
| Hadzic et al. (2013) | **no direct effects** | n/a | **indirect effects** linking 1–15 working hours to more PB and more than 40 hours as well as not working for pay to less PB via maternal parenting practices; no further association |
| Herbst (2017) | **no associations** | n/a | n/a |
| Lekfuangfu et al. (2015) | **association found** at child age 81 months linking returning to work at 7–12 months postpartum to less PB (no further associations) | n/a | n/a |
| Lombardi & Coley (2014) | **association found** linking returning to employment before 9 months part-time to lower PB (compared to no return to employment), no further associations | ethnicity did not moderate association, no interaction effects of return to work (before 9 months, between 9 and 24 months) and hours in nonparental care, maternal wages, depressive symptoms, and non-maternal household income (here, a main effect emerged linking return to employment before 9 months to lower PB compared to no return to employment) | inclusion of hours in nonparental care, maternal wages, and depressive symptoms did not alter results |
| Nomaguchi (2006) | 2 years: **no association**  4 years: cross-sectional **association found** linking part-time to more PB, no longitudinal associations^a^ | n/a | 2 years: inclusion of potentially mediating variables, i.e., mother-child interaction, child care, hours in school settings, did not alter results  4 years: cross-sectionally, full-time and part-time linked to more PB; longitudinally, results did not change |
| Pekkurnaz (2014) | **association found** linking more working hours to more PB | n/a | n/a |
| Perry & Fantuzzo (2010) | **association found** for PB linking longer lasting unemployment to less PB;  **no association** found for cooperative behavior | n/a | n/a |

| **Study** | **General findings** | **Moderator/subgroup analyses** | **Mediator analyses** |
| --- | --- | --- | --- |
| Philipsen Hetzner (2011) | **no associations** in both OLS regression models with and without propensity score matching | n/a | **direct effect** (with inclusion of various mediating variables, i.e., quality of home environment, attachment, child care, child well visits, maternal sensitivity, income, and time spent with child) linking full-time work to more PB |

n/a = not available/applicable; PB = prosocial behavior; OLS = ordinary least squares.

^a^In further, but unadjusted analyses no differences between part-time/part year employment full-time year round employment emerged.

| **Supplementary Table S8.** Results of moderator analyses. | | | | | | | | | |
| --- | --- | --- | --- | --- | --- | --- | --- | --- | --- |
|  | | | | | | | | | |
| **Employment status** | | | | | | | | | |
|  | **Study-level characteristics** | | | | **Sample-level characteristics** | | | | |
|  | Data source | Design | Adjustment | Risk of bias | Child age | Child sex | Timing of employment | Ethnicity | Marital status |
| Overall BP | ***Q* (6) = 32.27, *P*< .001** | *Q* (2) = 0.26, *P*= .880 | *Q* (1) = 0.63, *P* = .426 | *Q* (2) = 0.03, *P*= .986 | *Q* (1) = 1.17, *P*= .279 | *Q* (2) = 1.38, *P*= .502 | *Q* (1) = 0.08, *P*= .775 | ***Q* (3) = 8.36,**  ***P* < .05** | *Q* (3) = 0.38, *P*= .944 |
| Overall EBP | *Q* (5) = 7.37, *P*= .194 | n/a | *Q* (1) = 2.99, *P*= .08 | *Q* (1) = 0.23, *P*= .634 | *Q* (1) = 0.00, *P*= .990 | *Q* (2) = 4.29, *P*= .117 | *Q* (1) = 0.90, *P*= .343 | *Q* (3) = 7.32, *P*= .06 | *Q* (1) = 0.04, *P*= .838 |
| Aggression | n/a | *Q* (1) = 0.36, *P*= .550 | n/a | n/a | *Q* (1) = 0.66, *P*= .418 | n/a | *Q* (1) = 0.513, *P*= .130 | n/a | n/a |
| Hyperactivity/  inattention | n/a | n/a | n/a | n/a | n/a | n/a | n/a | n/a | n/a |
| Conduct problems | n/a | n/a | n/a | n/a | n/a | n/a | n/a | n/a | n/a |
| Overall IBP | n/a | n/a | n/a | n/a | n/a | n/a | n/a | n/a | n/a |
| Anxiety and depressive symptoms | n/a | n/a | n/a | n/a | n/a | n/a | n/a | n/a | n/a |
| PB | *Q* (2) = 0.36, *P*= .834 | *Q* (1) = 0.00, *P*= .979 | *Q* (1) = 0.47, *P*= .495 | *Q* (1) = 0.52, *P*= .473 | *Q* (1) = 0.02, *P*= .894 | n/a | *Q* (1) = 0.21, *P*= .645 | *Q* (1) = 0.47, *P*= .789 | ***Q* (1) = 3.96, *P*< .05** |
| **Full-time vs. part-time employment** | | | | | | | | | |
|  | **Study-level characteristics** | | | | **Sample-level characteristics** | | | | |
|  | Data source | Design | Adjustment | Risk of bias | Child age | Child sex | Timing of employment | Ethnicity | Marital status |
| Overall BP | n/a | n/a | ***Q* (1) = 6.30, *P*< .05** | *Q* (1) = 0.63, *P*= .427 | *Q* (1) = 0.09, *P* = .759 | n/a | ***Q* (1) = 6.30, *P*< .05** | n/a | *Q* (1) = 2.84, *P*= .092 |
| Overall EBP | n/a | *Q* (1) = 0.01, *P*= .918 | *Q* (1) = 0.02, *P*= .876 | *Q* (1) = 0.00, *P*= .960 | *Q* (1) = 0.00, *P*= .967 | n/a | *Q* (1) = 0.00, *P*= .950 | n/a | *Q* (1) = 2.53, *P*= .111 |
| Hyperactivity/  inattention | n/a | n/a | n/a | n/a | n/a | n/a | n/a | n/a | n/a |
| Overall IBP | n/a | n/a | n/a | n/a | n/a | n/a | n/a | n/a | n/a |
| PB | n/a | n/a | n/a | n/a | n/a | n/a | n/a | n/a | n/a |
| Important moderators appear in bold. For employment duration and return to work no moderator analyses were conducted. BP = behavior problems; EBP = externalizing behavior problems: n/a = not available/applicable; IPB = internalizing behavior problems; PB = prosocial behavior. | | | | | | | | | |

Corresponding author: Susan Garthus-Niegel

susan.garthus-niegel@ukdd.de

Technische Universität Dresden, Faculty of Medicine Carl Gustav Carus, Institute and Policlinic of Occupational and Social Medicine, Dresden, Germany; Medical School Hamburg, Institute for Systems Medicine (ISM) and Faculty of Medicine, Hamburg, Germany; Norwegian Institute of Public Health, Department of Childhood and Families, Oslo, Norway
